# Temporally evolving neuronal remodeling via the CTNNB1–NRG3–ERBB4 axis drives therapy resistance in glioblastoma

**DOI:** 10.1186/s43556-026-00638-7

**Published:** 2026-11-06

**Authors:** Sung Soo Kim, Ji Yoon Lee, Harim Koo, Zheng Yuan, Kyung-Hee Kim, Hyung Joon Kwon, Se Whan Park, Sun Il Choi, Ho-Shin Gwak, Heon Yoo, Young Taek Oh, Yong-Seok Oh, Antonio Iavarone, Seong-Min Park, Jason K. Sa, Jong Bae Park

**Affiliations:** 1 Neuroscience Institution, Lithuanian University of Health Sciences, Kaunas, Lithuania; 2 Department of Biomedical Sciences, Korea University College of Medicine, Seoul, Republic of Korea; 3 Department of Medical Science Convergence, Graduate School of Medical Science, University of Ulsan, Ulsan, Korea; 4 Henan and Macquarie University Joint Centre for Biomedical Innovation, School of Life Sciences, Henan University, Kaifeng, Henan China; 5 Neuro-Oncology Clinic, National Cancer Center, Goyang, Republic of Korea; 6 Department of Biological Sciences, College of Natural Sciences, Kangwon National University, ChuncheonKangwon, Republic of Korea; 7 Department of Brain-Cognitive Science, Daegu-Gyeongbuk Institute of Science and Technology (DGIST), Daegu, Republic of Korea; 8 Sylvester Comprehensive Cancer Center, University of Miami Miller School of Medicine, Miami, FL 33136 USA; 9 Department of Biochemistry and Molecular Biology, School of Medicine, Kyung Hee University, Seoul, Republic of Korea; 10 OmixAI Inc., Seoul, Republic of Korea

**Keywords:** Glioblastoma, Therapy resistance, NRG3, Temporal, Evolving, Neuronal

## Abstract

Glioblastoma (GBM) remains highly lethal despite early therapeutic responses, as resistance inevitably emerges. While clinical studies show improvements in progression-free survival (PFS) during the early response (ER) phase, the molecular trajectory bridging ER and recurrent resistance (RR)—and the adaptive resistance programs temporally engaged during this transition— remain poorly understood. To dissect the dynamic molecular evolution underlying therapy resistance, patient-derived xenograft (PDX) models were treated with anti-VEGF therapy. Longitudinal proteogenomic profiling across multiple time points was performed to capture time-resolved molecular changes associated with the tumor response to the therapy. Longitudinal analyses revealed a dynamic, temporally evolving response trajectory, with an initial response phase (IRP) marked by neuronal-like remodeling, and a late response phase (LRP) characterized by mesenchymal transdifferentiation and metabolic adaptation. Proteomic and phosphoproteomic analyses identified the CTNNB1–NRG3–ERBB4 signaling axis as a key driver of neuronal-like remodeling during the IRP. Mechanistically, β-catenin activation upregulated NRG3, promoting postsynaptic scaffold remodeling, while pharmacological inhibition of β-catenin/CBP with PRI-724 disrupted this neuronal program and significantly extended survival in vivo. These findings place neuronal-like remodeling within a time-resolved framework of tumor adaptation to anti-VEGF therapy in GBM and highlight the CTNNB1–NRG3–ERBB4 axis as a tractable therapeutic target, providing a rationale for combination strategies to overcome anti-VEGF therapy failure.

**Supplementary Information:**

The online version contains supplementary material available at https://doi.org/10.1186/s43556-026-00638-7.

## Introduction

GBM is the most common and aggressive brain tumor in adults, with a median survival of less than 15 months due to frequent recurrences [1]. Various therapeutic strategies, including standard chemoradiation and targeted therapies, have shown improvements in PFS without corresponding gains in overall survival (OS), highlighting the persistent challenge of adaptive resistance emerging during the transition from PFS to OS in both primary and recurrent GBM [2, 3]. Notably, resistance-associated processes such as hypoxia, epithelial–mesenchymal transition (EMT), and metabolic rewiring are shared across multiple therapeutic modalities, rather than being unique to any single intervention, suggesting shared adaptive programs that limit durable responses [2–7].

Prominent histopathological characteristics of GBMs consist of infiltrative growth, pleomorphic vessels with a necrotic phenotype, and rapid vascularization, driven by aberrant activation of the vascular endothelial growth factor (VEGF) pathway [4]. anti-VEGF therapy represent both clinically validated interventions and experimentally tractable models for elucidating mechanisms of adaptive resistance in GBM [5]. In this context, the therapy initially reduces tumor burden, transiently improving PFS, yet tumors frequently rebound without OS benefit, thereby providing a clearly traceable treatment-response trajectories. This pattern provides a tractable temporal window to study how adaptive resistance emerges during treatment. Importantly, the same RR mechanisms observed across multiple standard therapies—hypoxia-driven remodeling, EMT, and metabolic plasticity—also emerge during VEGF blockade [2–7].

Despite these insights, longitudinal molecular programs linking ER to RR remain poorly defined, representing a critical gap in understanding the temporal evolution of therapy resistance. To address this, we employed PDX models under anti-VEGF therapy as a clinically relevant system to capture the temporal evolution of adaptive resistance [8–17]. By performing longitudinal proteogenomic profiling at multiple defined time points, we were able to capture the continuous, time-resolved narrative of tumor adaptation occurring in the IRP and LRP in the PDX models. This integrative, longitudinal approach enables mapping of dynamic oncogenic signaling networks and cellular states, revealing shared adaptive resistance programs that may extend beyond anti-VEGF therapy to other treatment modalities [18–22].

Our longitudinal proteogenomic analyses revealed a clearly delineated, time-resolved trajectory of resistance. During the IRP, tumors exhibited a neuronal-like remodeling, characterized by activation of synaptogenic signaling and WNT/β-catenin pathways, with prominent engagement of the CTNNB1–NRG3–ERBB4 signaling axis. These early molecular events likely facilitate rapid adaptation to therapeutic stress, providing a transient survival advantage. As treatment progressed, this neuronal-like remodeling declined, giving rise to the LRP marked by mesenchymal transdifferentiation and metabolic reprogramming, reflecting full engagement of the tumor microenvironment and the establishment of durable therapy resistance.

Collectively, these observations indicate that GBM employs a temporally organized, evolving adaptive strategy, initially co-opting neuronal-like remodeling to withstand acute therapeutic pressure, followed by mesenchymal and microenvironmental reprograms to sustain long-term resistance. This framework highlights the CTNNB1–NRG3–ERBB4 axis as a tractable therapeutic target and provides a rationale for interventions aimed at disrupting adaptive transitions to improve outcomes in GBM therapy. Overall, this study provides a novel, time-resolved framework for understanding evolving adaptive resistance trajectories in GBM.

## Results

### Characterization of IRP and LRP in longitudinally monitored orthotopic PDX models treated with anti-VEGF Therapy

**Fig. 1:**
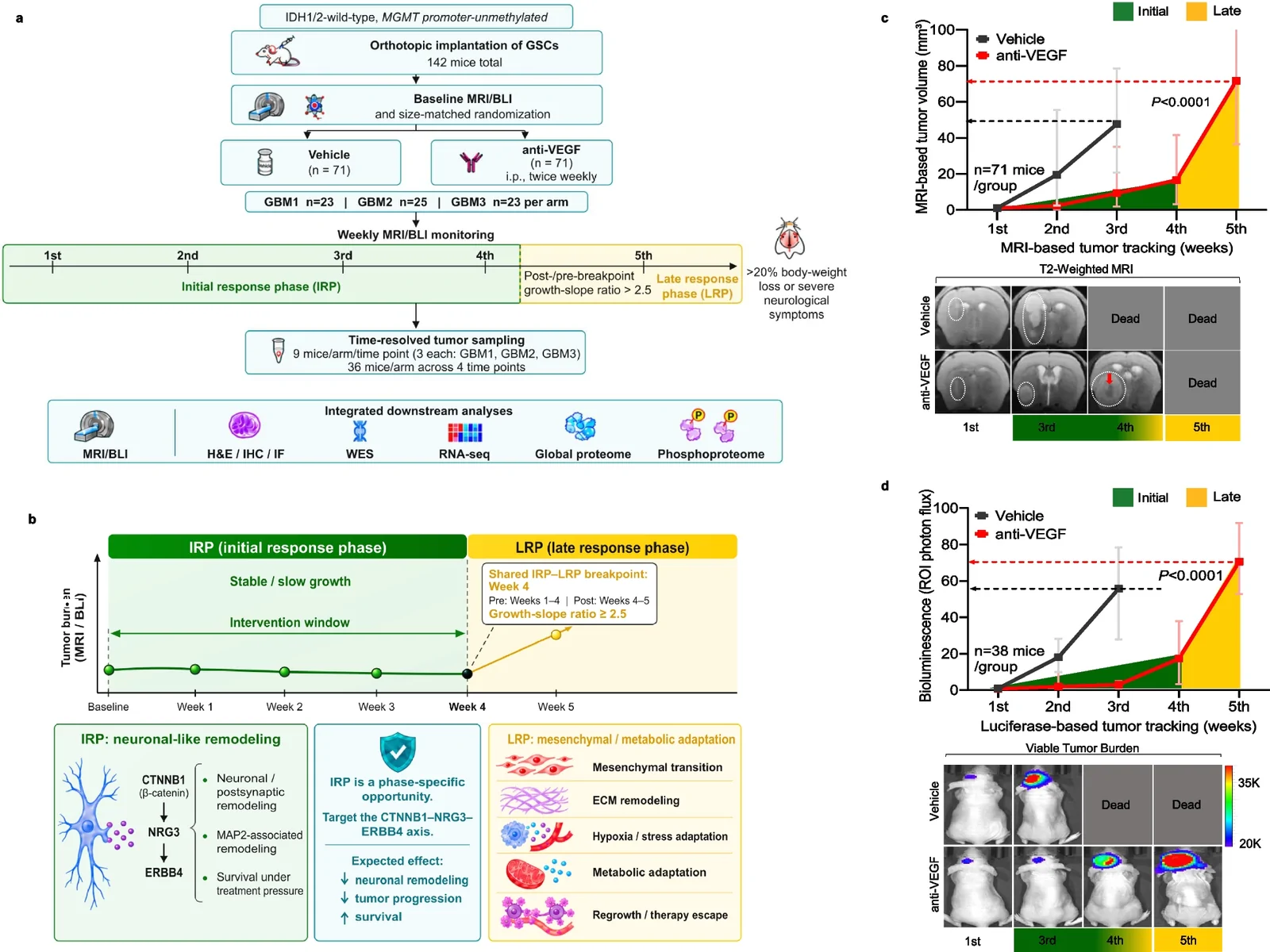
Establishment of longitudinal orthotopic PDX models reveals biphasic response dynamics comprising the IRP and LRP during anti-VEGF therapy. **a** Experimental workflow for longitudinal orthotopic GBM PDX modeling (IDH1/2-wild-type and MGMT promoter-unmethylated GBM1, GBM2, GBM3, and GBM4 models). Patient-derived GSCs were orthotopically implanted into immunocompromised mice. **b** Schematic summary of the IRP/LRP adaptive trajectory and potential intervention opportunity during anti-VEGF therapy in glioblastoma. Orthotopic GBM/PDX tumors initially exhibit an initial response phase (IRP; weeks 1–4) characterized. At the fifth imaging time point, tumors were assigned to the late response phase (LRP). **c** Longitudinal T2-Weighted MRI-derived tumor volumes demonstrate a reproducible biphasic response to anti-VEGF therapy with IRP (green) and LRP (yellow) indicated. The IRP was defined by tumor stabilization/regression, whereas the LRP was characterized by rapid tumor regrowth. hicle-treated tumors progressed without a stabilization phase and reached humane endpoint criteria earlier than anti-VEGF-treated tumors (vehicle, *n* = 71; anti-VEGF, *n* = 71, composed of GBM1 (*n* = 23), GBM2 (*n* = 25), and GBM3 (*n* = 23) PDX tumors per treatment arm). Dotted outlines indicate tumor regions on representative MRI images, and red arrows indicate areas consistent with necrotic change. “Dead” denotes animals that reached predefined humane endpoint criteria prior to the corresponding imaging time point. Statistical significance was assessed using a two-way mixed-effects analysis followed by Šídák’s multiple-comparison test. **d** Longitudinal validation of viable tumor burden in orthotopic GBM-Luc tumors during anti-VEGF therapy. Left, ROI-based quantification of BLI signal over sequential longitudinal time points in vehicle and anti-VEGF–treated cohorts, with IRP (green) and LRP (yellow) indicated. The boundary of IRP and LRP was annotated using the same operational criterion applied to MRI (ANOVA, *p* < 0.0001; vehicle n = 38, anti-VEGF *n* = 38, composed of GBM1 (*n* = 9), GBM2 (*n* = 19), and GBM3 (n = 8) PDX tumors per treatment arm). Right, representative BLI provided an orthogonal readout of viable tumor burden to complement MRI-based volumetric assessment under anti-VEGF therapy. Statistical significance was assessed using a two-way mixed-effects analysis followed by Šídák’s multiple-comparison test

To model therapy resistance in GBM, we established PDX models by orthotopically implanting glioblastoma stem-like cells (GSCs) into immunocompromised mice [8–17]. One week after implantation, baseline tumor burden was quantified by MRI and bioluminescence imaging (BLI) signal intensity to baseline-match mice prior to treatment [18, 19]. Baseline-matched mice were then randomized to vehicle (*n* = 71) or anti-VEGF (*n* = 71) treatment (Fig. S1a). Mice that did not meet endpoint criteria at the time of tissue harvesting were excluded from survival analysis but were included in other assessments, including MRI, BLI, immunohistochemistry, and H&E staining. A longitudinal study design, coupled with a pre-specified statistical framework, ensured robust treatment-response evaluation. This framework enabled time-resolved tumor sampling for proteogenomic analysis (Fig. 1a).

The PDX models showed that all vehicle-treated mice uniformly developed endpoint-defining symptoms by the third week (3rd) after orthotopic implantation of GSCs. Figure 1b presents MRI-derived tumor volumes over time in anti-VEGF–treated groups. The first through fourth time points constituted the relatively shallow IRP growth segment, whereas the fourth-to-fifth time-point interval constituted the accelerated LRP growth segment. The post-breakpoint growth slope was at least 2.5-fold greater than the pre-breakpoint growth slope, satisfying the operational criterion for the LRP trajectory. Thus, the ≥ 2.5-fold value represented the increase in growth rate across the fourth-time-point breakpoint rather than a direct tumor-volume ratio. All orthotopic PDX models exhibited temporally two distinct response phases to anti-VEGF therapy. The IRP was defined as tumor stabilization or regression, whereas the LRP was characterized by rapid tumor regrowth, mimicking RR in human GBM (radiographic/clinical progression after an early period of disease control) [18, 19]. Compared with IRP tumors, LRP tumors displayed more prominent necrotic and hypoxia-associated features, including increased HIF-1α staining and corresponding T2-Weighted MRI and H&E histological changes, consistent with accelerated regrowth under anti-VEGF treatment [4, 5] (Fig. 1b and Fig. S1b).

To strengthen treatment-response assessment beyond T2-Weighted MRI-based volumetric analysis, we employed an orthotopic GBM-Luciferase (GBM-Luc) reporter system for time-resolved in vivo quantification of viable tumor burden by BLI [13]. Mice bearing orthotopic GBM-Luc tumors were treated with vehicle or anti-VEGF therapy. anti-VEGF produced an initial stabilization or suppression of tumor burden, followed by tumor rebound, thereby recapitulating the distinction between IRP and LRP defined by MRI in Fig. 1b. ROI-based BLI analysis showed a marked increase in viable tumor burden between the fourth and fifth time points. BLI was used as an orthogonal readout of viable tumor burden and was not used independently to define the IRP/LRP boundary, which was determined from the MRI-derived growth-slope ratio (Fig. 1c).

Together, longitudinal BLI corroborates the time-resolved response trajectory observed in PDX models and provides an orthogonal readout to complement MRI under therapy.

### Integrative proteo-transcriptomic profiling reveals WNT-associated neuronal-like remodeling in the IRP

To elucidate time-resolved molecular programs underlying therapy resistance, we performed longitudinal multi-omics profiling across MRI/BLI-defined IRP and LRP. This design enabled time-resolved mapping of multilayer molecular changes across IRP and LRP response states (Fig. 1a).

**Fig. 2:**
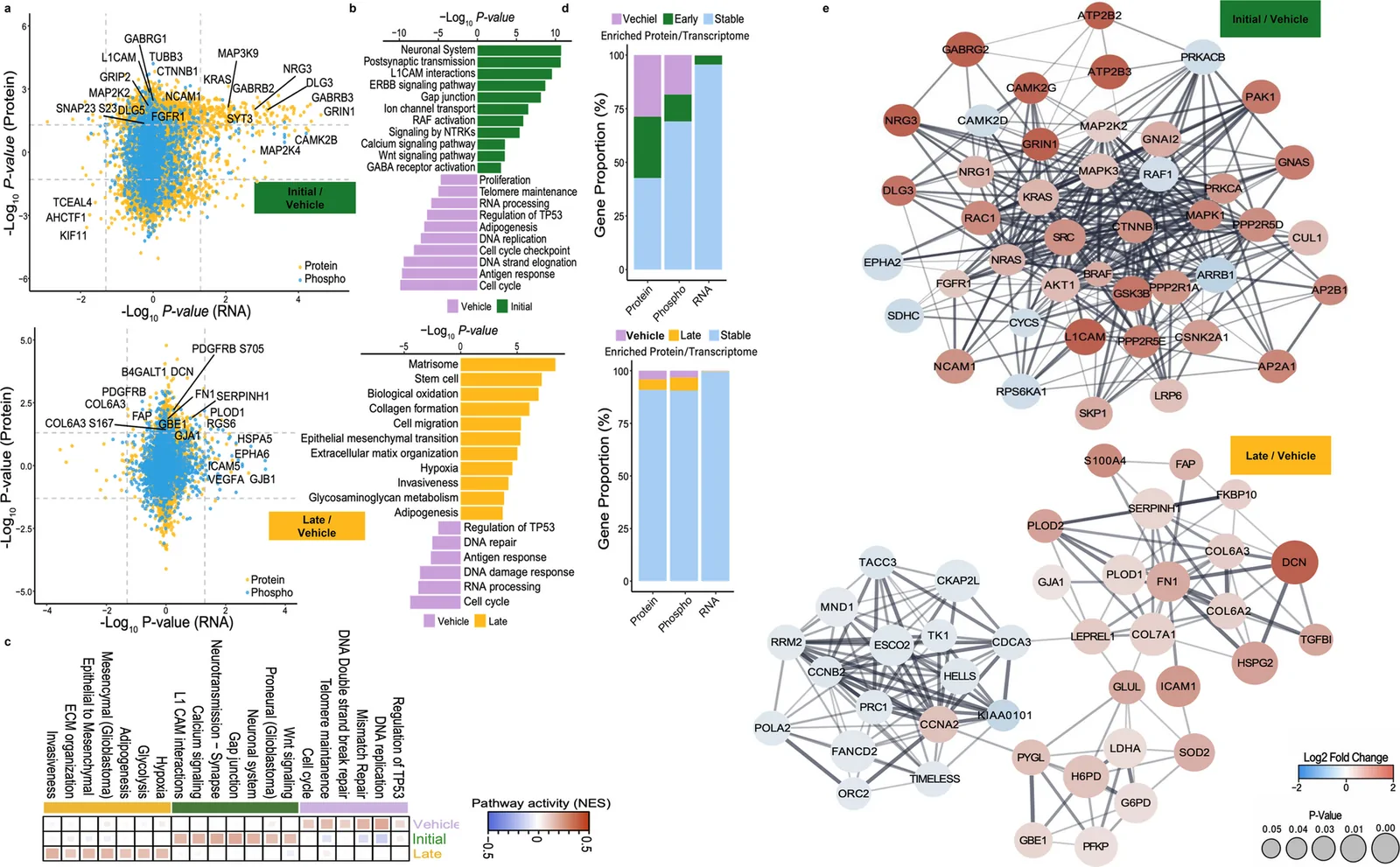
Integrative proteo-transcriptomic profiling reveals WNT-associated neuronal-like remodeling in the IRP. **a** Proteogenomic analyses included nine mice per treatment condition at each time point (n = 9 mice; three mice each for GBM1, GBM2, and GBM3). Tissues from the three mice within each PDX model were generating three independent model-level samples per condition (n = 3). Across four time points, 12 mice were used per PDX model and 36 mice per treatment condition. Upper, Integrated proteomic comparison of IRP tumors versus vehicle controls. Left, scatter plot comparing transcript-level significance (x-axis) with protein-level significance (y-axis), highlighting molecules preferentially enriched at the protein/phosphoprotein level during the IRP. Representative IRP-associated neuronal/WNT-related molecules. Lower, Integrated proteomic comparison of LRP tumors versus vehicle controls. Left, scatter plot comparing transcript-level significance (x-axis) with protein-level significance (y-axis), highlighting LRP-associated molecules enriched preferentially at the protein/phosphoprotein level. Nominal P values are shown for visualization, whereas statistical significance was defined using FDR-adjusted q values, with q < 0.05 considered significant. **b** Upper, Pathway enrichment analysis of IRP-enriched and IRP-depleted proteins/phosphoproteins. Lower, pathway enrichment analysis of LRP-enriched and LRP-depleted proteins/phosphoproteins. Bar length represents − log10 nominal *P* value for visualization, whereas statistical significance was defined using Benjamini–Hochberg FDR-adjusted q values, with q < 0.05 considered significant. **C** Heatmap showing pathway activity scores vehicle, IRP, and LRP tumors. Color intensity indicates normalized enrichment score (NES) or relative pathway activity derived from predefined pathway gene sets from proteomic and phosphoproteomic datasets. Pathway-level significance was assessed using the enrichment statistics generated by the corresponding pathway analysis, with multiple testing controlled by Benjamini–Hochberg FDR correction where applicable. **d** Upper, relative proportions of enriched and stable features across proteomic, phosphoproteomic, and transcriptomic datasets in IRP versus vehicle comparisons. Lower, relative proportions of enriched and stable features across proteomic, phosphoproteomic, and transcriptomic datasets in LRP versus vehicle comparisons. Feature categories were defined using the same FDR-adjusted significance thresholds applied in panels A. **e** Upper, PPI network constructed from IRP-enriched proteomic and phosphoproteomic datasets relative to vehicle controls. Node color indicates relative abundance change (red, upregulated; blue, downregulated), node size reflects statistical significance (P value), and edge thickness represents interaction confidence. CTNNB1, FGFR1, and NRG3 emerged as highly connected nodes within a broader neuronal/WNT-associated network. Lower, PPI network constructed from LRP-associated proteomic and phosphoproteomic datasets relative to vehicle controls. The LRP network is dominated by mesenchymal-, extracellular matrix-, and metabolic-remodeling proteins, whereas a distinct cell-cycle–associated cluster is relatively depleted. Together, these networks support a phase-dependent transition from an IRP-associated neuronal/WNT adaptive state to an LRP-associated mesenchymal and ECM-remodeling state. Node selection was based on the FDR-adjusted differential omics analysis, whereas node size shows nominal P value for visualization and edge thickness shows interaction confidence

To uncover key biological processes underlying the tumor response to anti-VEGF treatment, we integrated transcriptomic, proteomic, and phosphoproteomic datasets to assess whether protein-level regulation more directly captures therapy-associated changes beyond transcript abundance. Although a several number of proteins and phosphoproteins showed changes concordant with transcriptomic levels, substantial key molecules exhibited discordant regulation, suggesting a prominent role for post-transcriptional and post-translational mechanisms. During the IRP, we observed selective protein-level enrichment of a coordinated neuronal-like and postsynaptic remodeling program comprising WNT/β-catenin signaling, neuregulin–ERBB signaling, GABAergic/synaptic components, and MAPK-associated signaling molecules, including CTNNB1, NRG3, ERBB4, FGFR1, GABRG1, GABRB2, GABRB3, GRIP2, SNAP23, MAP2K2, MAP2K4, and MAP3K9 [20–25]— preferentially at the protein level (Fig. 2a). Pathway enrichment analysis further supported this module-level interpretation, revealing coordinated activation of synaptic, ERBB/neuregulin, MAPK/RAS-RAF, and WNT/β-catenin-associated programs during the IRP [20–23] (Fig. 2b). In contrast, LRP tumors exhibited increased protein and phosphoprotein abundance of mesenchymal, stromal, and ECM-remodeling molecules, including B4GALT1, DCN (decorin), FN1 (fibronectin1), PDGFRB, COL6A3, FAP, SERPINH1, and PLOD1, together with stress-, metabolic-, and vascular-associated proteins such as HSPA5, GBE1, and VEGFA [26–30]. These molecules are associated with glycosaminoglycan biosynthesis, collagen processing, extracellular matrix organization, hypoxia/stress adaptation, and mesenchymal remodeling [30–35]. Meanwhile, both IRP and LRP tumors showed decreased levels of proteins involved in DNA replication, mismatch repair, and cell cycle regulation (Fig. 2c). Quantitative comparison of multi-omic signatures further revealed that protein and phosphoprotein profiles underwent more substantial shifts than transcriptomic profiles under treatment pressure (Fig. 2d). These results highlight the value of proteomic data in identifying rapid, functionally relevant drivers of resistance.

To identify potential therapeutic targets driving the early neuronal-like remodeling during anti-VEGF treatment, we constructed a protein–protein interaction (PPI) network by integrating proteomics and phosphoproteomics datasets to generate an interactome of proteins ranked according to their connectivity within cellular signaling networks. Notably, CTNNB1, L1CAM, GABR2, NRG3, and DLG3 emerged among the most highly connected nodes within neuronal-like and WNT-associated networks, suggesting their potential roles in treatment resistance (Fig. 2e). CTNNB1, NRG3, DLG family genes, and broader neuron-associated signaling markers represent key components of neurotrophic and synaptic pathways, where ligand–receptor interactions activate intracellular signaling cascades associated with neuronal development and synaptic function. Within this network, CTNNB1 was prioritized based on convergent pathway- and network-level evidence. NRG3 was concurrently identified as a prominent network-associated ligand, and ERBB4 represented its cognate receptor within the IRP-associated neuronal-like signaling program [36–43]. Together, these findings supported the prioritization of the CTNNB1-linked NRG3–ERBB4 signaling axis for subsequent functional investigation. When we applied the same analytical framework to LRP tumors, we observed enrichment of mesenchymal-associated and ECM remodeling-associated proteins, including POSTN, ICAM1, DCN, FN1, PLOD2, SERPINH1, TGFBI, and S100A4 [43–45].

In summary, this integrative multi-omic analysis indicates that anti-VEGF therapy resistance in GBM is temporally structured: IRP adaptation involves WNT/neuronal-like remodeling, whereas the LRP is characterized by mesenchymal and ECM-driven remodeling, highlighting phase-specific vulnerabilities for therapeutic intervention.

### Longitudinal proteomic profiling identifies temporally evolving molecular trajectories associated with anti-VEGF therapy resistance

**Fig. 3:**
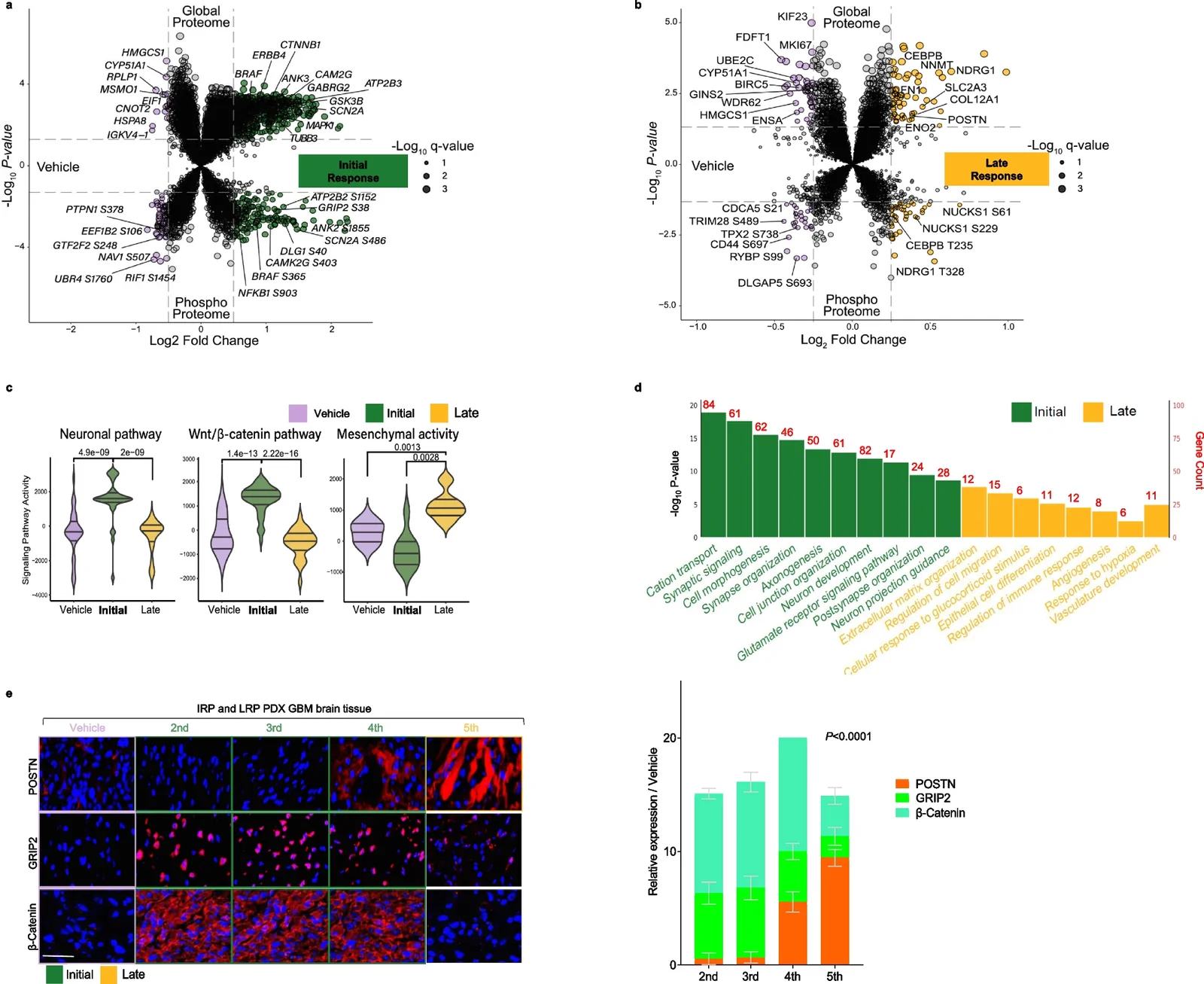
Longitudinal proteomic profiling identifies temporally evolving molecular trajectories associated with anti-VEGF therapy resistance. **a** Integrated volcano plot of the global proteome (upper panel) and phosphoproteome (lower panel) comparing IRP tumors with vehicle controls. The x-axis indicates log2 fold change, and the y-axis indicates − log10 nominal P value. Circle size represents − log10 Benjamini–Hochberg FDR-adjusted q value. Colored points indicate proteins or phosphosites meeting the indicated fold-change and FDR-adjusted significance thresholds. For proteomic and phosphoproteomic analyses, nominal P values were used for volcano plot visualization, whereas FDR-adjusted q values were used to define statistical significance. Unless otherwise indicated, significance was defined as FDR-adjusted q < 0.05. **b** Integrated volcano plot of the global proteome (upper panel) and phosphoproteome (lower panel) comparing LRP tumors with vehicle controls. The x-axis indicates log2 fold change, and the y-axis indicates − log10 nominal P value. Circle size represents − log10 Benjamini–Hochberg FDR-adjusted q value. Colored points indicate proteins or phosphosites meeting the indicated fold-change and FDR-adjusted significance thresholds. For proteomic and phosphoproteomic analyses, nominal P values were used for volcano plot visualization, whereas FDR-adjusted q values were used to define statistical significance. Unless otherwise indicated, significance was defined as FDR-adjusted q < 0.05. **c** Violin plots showing pathway activity scores for the neuronal pathway. Pathway activity scores were calculated from predefined pathway gene/protein sets and compared among groups. Statistical significance was assessed using Kruskal–Wallis test followed by pairwise Wilcoxon rank-sum tests, with Benjamini–Hochberg FDR correction applied across tested pathways where applicable. **d** GO enrichment analysis of differentially expressed proteomic features in IRP and LRP tumors. GO enrichment was performed using proteins/phosphoproteins passing the differential abundance criteria defined in panels A,B. Bar height indicates enrichment strength (gene counts), and the overlaid line indicates statistical significance as indicated in the plot. Multiple testing was corrected using the Benjamini–Hochberg FDR method. The corresponding gene count is indicated by red-colored numerical labels positioned above each bar. **e**. Left, representative temporal immunofluorescence images of therapy-treated orthotopic GBM2 PDX tumors collected across sequential treatment cycles (vehicle, 2nd, 3rd, 4th, and 5th time points). Right, quantitative analysis across three independent GBM PDX models confirmed a decrease in CTNNB1 and GRIP2 expression with a concomitant increase in POSTN during progression from IRP to LRP. Scale bar, 50 μm. Data are presented as mean ± SEM. Statistical significance was determined using one-way ANOVA followed by Tukey’s post hoc test

Building on the proteo-transcriptomic analysis, which indicated that key therapy-associated signals were preferentially captured at the protein levels beyond transcript abundance, we next focused on the global proteome and phosphoproteome to define phase-specific molecular trajectories across IRP and LRP tumors. Volcano plot analysis highlighted distinct IRP- and LRP-associated molecular features relative to vehicle controls. IRP tumors showed increased abundance of WNT/β-catenin pathway components (CTNNB1, GSK3B) and synapse-associated proteins (DLG1, GABRG2, GRIP2, ERBB4) [20–25, 46], consistent with activation of neuronal/synaptic-like remodeling during IRP (Fig. 3a). In contrast, LRP tumors displayed significant upregulation of EMT-associated markers (CEBPB, POSTN) and hypoxia-responsive proteins (NDRG1, SLC2A3) linked to mesenchymal transition and microenvironmental remodeling in GBM [47, 48] (Fig. 3b).

To delineate the biological implications of these proteomic alterations, we first performed signaling pathway activity analysis. This revealed that neuronal development and WNT/β-catenin signaling were selectively activated in IRP tumors, whereas mesenchymal-associated pathways were preferentially enriched in LRP tumors (Fig. 3c). We next conducted Gene Ontology (GO) enrichment analysis, which showed that IRP tumors were significantly enriched for biological processes related to neuronal development and synaptic signaling, while LRP tumors exhibited enrichment of pathways associated with angiogenesis, cell migration, and extracellular matrix organization (Fig. 3d).

To corroborate the temporally evolving resistance trajectory identified by longitudinal proteomic analyses. We performed temporal immunofluorescence analysis in therapy-treated orthotopic PDX mouse brains, revealing robust WNT pathway activation during the IRP. Across sequential treatment cycles, IRP-associated markers—including β-catenin (CTNNB1), a central effector of WNT signaling, as well as the neuronal-associated protein GRIP2—were markedly upregulated at early time points and remained elevated through intermediate stages (Fig. 3e). At later time points corresponding to the transition toward the LRP phase and tumor regrowth, expression of these IRP-associated markers declined, accompanied by a pronounced increase in POSTN, an extracellular matrix protein associated with mesenchymal reprogramming and invasive tumor behavior.

This temporal shift recapitulates the transition from neuronal-like to mesenchymal remodeling observed in the proteomic analyses, reinforcing a dynamic phenotypic reprogramming trajectory underlying therapy resistance.

### CTNNB1–NRG3–ERBB4 signaling drives IRP-associated neuronal-like remodeling and tumor cell expansion

**Fig. 4:**
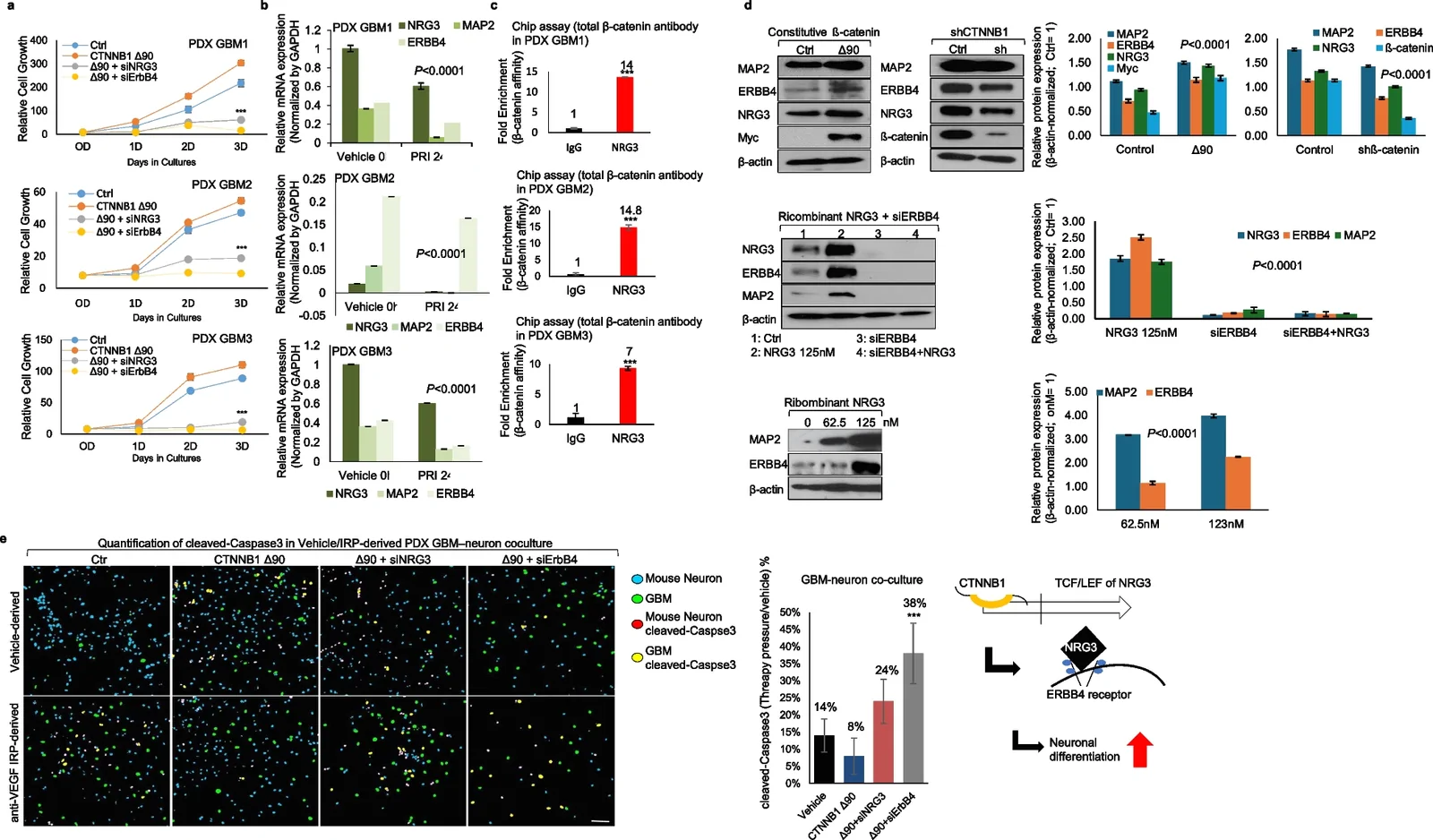
CTNNB1–NRG3–ERBB4 signaling promotes IRP-associated neuronal-like remodeling and GBM cell survival under therapeutic stress. **a** Growth analysis of GBM1-3 cells with constitutive β-catenin activation or CTNNB1 suppression. CTNNB1-Δ90 expression increased GBM cell expansion. In CTNNB1-activated cells, siRNA-mediated silencing of NRG3 or ERBB4 attenuated the proliferative advantage, indicating that CTNNB1-driven tumor cell expansion depends. Data are presented as mean ± SEM. Statistical significance was determined using one-way ANOVA followed by Tukey’s post hoc test. **b** qPCR analysis of NRG3, MAP2, and ERBB4 expression in primary GBM1-3 cells after PRI-724 treatment. mRNA levels were normalized to GAPDH and expressed relative to vehicle controls. PRI-724 reduced NRG3, MAP2, and ERBB4 expression, consistent with suppression of the neuronal-associated adaptive program. Data are mean ± SEM; one-way ANOVA with Tukey’s post hoc test**c** β-catenin chromatin immunoprecipitation analysis at the NRG3 regulatory region. β-catenin binding was enriched at the NRG3 locus compared with IgG control, supporting direct transcriptional regulation of NRG3 by β-catenin. Data are presented as mean ± SEM. Statistical significance was determined using one-way ANOVA followed by Tukey’s post hoc test. **d** Upper, representative immunoblot analysis validating NRG3–ERBB4–MAP2 pathway connectivity in GBM2 cells. Recombinant NRG3 treatment increased ERBB4-associated signaling and induced MAP2 expression, whereas siRNA-mediated ERBB4 knockdown attenuated NRG3-induced MAP2 upregulation. Lower, Schematic model of the CTNNB1– NRG3–ERBB4 signaling hierarchy. β-catenin-dependent transcription induces NRG3 expression, secreted NRG3 activates ERBB4 signaling, and ERBB4 promotes MAP2-associated neuronal-like remodeling in GBM cells. Quantification Data are presented as mean ± SEM. Statistical significance was determined using one-way ANOVA followed by Tukey’s post hoc test. **e** Representative immunofluorescence images and quantification of cleaved caspase-3-positive GBM cells in co-cultures of GFP-labeled primary GBM cells and CFP-labeled mouse cortical neurons. GBM cells were isolated from vehicle-treated orthotopic tumors or from in vivo anti-VEGF-treated tumors collected during the IRP, as indicated in the figure. CTNNB1-Δ90 expression reduced cleaved caspase-3 positivity in anti-VEGF IRP-derived GBM cells, whereas NRG3 or ERBB4 silencing reversed this effect and increased apoptotic sensitivity. Data are presented as mean ± SEM from three independent experiments. Statistical significance was assessed using one-way ANOVA followed by Tukey’s multiple-comparison test

To assess the role of WNT/β-catenin signaling in promoting proliferative advantage through the CTNNB1–NRG3–ERBB4 axis during the IRP, we generated GBM tumor cell lines with either constitutive activation of β-catenin (CTNNB1-Δ90) [49] or shRNA-mediated CTNNB1 knockdown. CTNNB1-Δ90 significantly increased GBM cell proliferation, whereas siRNA-mediated silencing of NRG3 or ERBB4 attenuated this proliferative advantage in CTNNB1-Δ90-activated cells (Fig. 4a and S2a). These results indicate that CTNNB1-driven tumor cell expansion requires downstream NRG3–ERBB4 signaling (Fig. S2b).

To determine whether β-catenin regulates the neuronal-like remodeling, we treated GBM cells with PRI-724, a pharmacological inhibitor of β-catenin/CBP-dependent transcription [50–52]. PRI-724 treatment reduced mRNA expression of NRG3, ERBB4, and MAP2, indicating that β-catenin transcriptional activity is required for induction of this neuronal-associated signaling (Fig. 4b). Consistent with this, chromatin immunoprecipitation analysis demonstrated marked enrichment of β-catenin binding at the NRG3 regulatory region compared with IgG control, supporting direct transcriptional regulation of NRG3 by β-catenin (Fig. 4c).

To validate functional connectivity within the CTNNB1–NRG3–ERBB4 axis, GBM cells were treated with recombinant NRG3 ligand. Western blot analysis showed that recombinant NRG3 treatment enhanced ERBB4-associated neuronal signaling, as evidenced by MAP2 induction, whereas siRNA-mediated ERBB4 silencing abolished this response (Fig. 4d). Together, these findings support a signaling hierarchy in which β-catenin transcriptionally induces NRG3, NRG3 activates ERBB4, and ERBB4 signaling promotes MAP2-associated neuronal-like remodeling.

To determine whether the IRP-associated neuronal/postsynaptic phenotype was enhanced in a neuron-containing environment, we established a co-culture system comprising GFP-labeled primary GBM cells and CFP-labeled mouse cortical neurons. This simplified assay was designed to assess tumor-cell postsynaptic scaffold-marker remodeling and survival in response to neuronal contact. CTNNB1-Δ90 expression increased postsynaptic scaffold-marker positivity and reduced cleaved caspase-3 positivity in GBM cells, whereas silencing NRG3 or ERBB4 attenuated these effects. These findings support a functional role for CTNNB1-driven NRG3–ERBB4 signaling in the neuron-associated adaptive phenotype of IRP-derived GBM cells. Apoptotic GBM cells were quantified by cleaved caspase-3 staining using predefined gating criteria: mouse cortical neurons had a nuclear area < 2 × 10⁸ nm^2^ and intensity > 13,700, while GBM-treated cells had a nuclear area > 2 × 10⁸ nm^2^ or intensity < 13,700. Cells with cleaved-Caspase3 staining intensity > 2,500 were classified as apoptotic (Fig. 4e). Overall, these findings define a functional CTNNB1-NRG3-ERBB4 signaling cascade that promotes GBM cell proliferation, survival, neuronal-like remodeling, and postsynaptic-like remodeling during the IRP.

### CTNNB1–NRG3–ERBB4 signaling promotes postsynaptic-like tumor–neuron interactions and survival under therapeutic stress

**Fig. 5:**
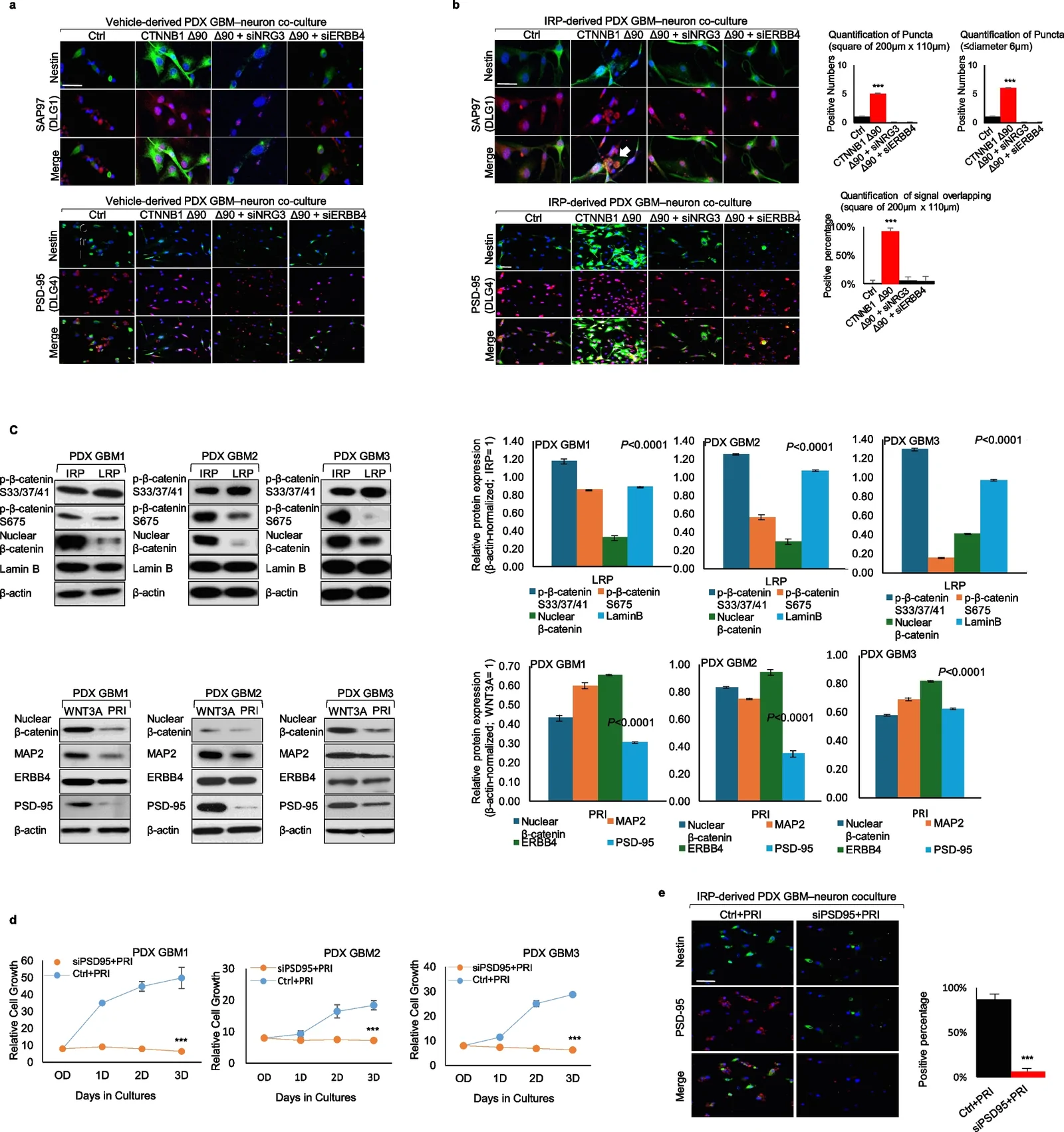
CTNNB1–NRG3–ERBB4 signaling promotes postsynaptic-like tumor–neuron interactions and survival under therapeutic stress. **a**. Representative immunofluorescence images of vehicle-derived PDX GBM2–mouse cortical neuron co-cultures stained for Nestin, SAP97/DLG1, and PSD95/DLG4. SAP97/DLG1 and PSD95/DLG4 were used as postsynaptic scaffold markers, and Nestin was used to define GSCs. Upper panels show low-density, high-magnification fields used to resolve discrete SAP97/DLG1-positive puncta at the single-cell level. SAP97/DLG1-positive puncta were counted within Nestin-positive GBM-cell regions, and punctate structures with a diameter ≤ 6 μm were included as discrete puncta rather than diffuse staining or nonspecific aggregates. Lower panels show broad-field, low-magnification views used to assess PSD95/DLG4-positive postsynaptic scaffold-marker signal across Nestin-positive GBM-cell regions. SAP97/PSD95 positivity was quantified within Nestin-positive GBM-cell regions to determine the fraction of GBM cells exhibiting postsynaptic scaffold-marker signal. Quantification was performed within GBM-cell-defined regions rather than across the mixed co-culture field. Scale bar, 50 μm. Data are no significance. **b** Representative immunofluorescence images of anti-VEGF–treated IRP-derived PDX GBM2–mouse cortical neuron co-cultures collected at the fourth treatment time point and stained. Nestin was used to define GSCs regions. Upper panels show high-magnification fields used to resolve discrete SAP97/DLG1-positive puncta within Nestin-positive GBM-cell regions. Lower panels show broad-field views used to assess PSD95/DLG4-positive postsynaptic scaffold-marker signals across Nestin-positive GBM-cell regions. The white arrow marks a representative punctate structure showing spatial overlap between SAP97/DLG1 or PSD95/DLG4 signal and a Nestin-positive GBM-cell region. Similar discrete, round punctate structures within Nestin-positive GBM-cell regions were counted as postsynaptic scaffold-marker-positive puncta. Scale bar, 50 μm. Upper graphs quantify the number of discrete SAP97/DLG1-positive puncta within standardized 200 × 110 μm Nestin-positive GBM-cell regions of interest. Puncta were defined as round structures ≤ 6 μm in diameter. Lower graphs quantify the percentage of PSD95/DLG4-positive signal overlapping with Nestin-positive GBM-cell regions. Anti-VEGF–treated IRP-derived PDX GBM cultures and CTNNB1-Δ90–expressing GBM cells showed increased postsynaptic scaffold-marker-positive puncta/overlap, whereas siNRG3 or siERBB4 reduced this phenotype. Data are mean ± SEM; one-way ANOVA with Tukey’s post hoc test. **c** Immunoblot analysis of β-catenin activation state and neuronal/postsynaptic-associated markers in IRP- and LRP-derived GBM1-3 cells. Phosphorylation of β-catenin at Ser33/Ser37/Thr41 was assessed as a degradation-associated modification, whereas phosphorylation at Ser675 and nuclear β-catenin accumulation were assessed as activation-linked readouts. IRP-associated GBM cells showed increased p-β-catenin Ser675 and nuclear β-catenin, together with increased ERBB4, MAP2, and PSD95 expression. PRI-724 (300 ng/mL for 48 h) treatment reduced β-catenin/CBP-dependent signaling and decreased ERBB4, MAP2, and PSD95 expression, whereas WNT3A (100 ng/mL for 48 h) stimulation enhanced β-catenin pathway activation. Quantification Data are presented as mean ± SEM. Statistical significance was determined using one-way ANOVA followed by Tukey’s post hoc test. **d** Growth/survival analysis of IRP-derived GBM cells under therapeutic stress (PRI 100 ng/mL for 48 h) following PSD95 silencing. Control IRP cells maintained progressive expansion and survival, whereas siRNA-mediated PSD95 silencing reduced the resistance-associated growth/survival advantage and shifted IRP-derived cells toward a therapy-sensitive phenotype. Data are presented as mean ± SEM. Statistical significance between groups was determined using one-way ANOVA followed by Tukey’s post hoc test. **e** Representative immunofluorescence images and quantification of PSD95-positive postsynaptic-associated signals in tumor–neuron co-culture. Control IRP-derived GBM2 cells formed diffuse PSD95-positive signals in the presence of neurons, whereas PSD95 silencing reduced signals formation and weakened postsynaptic-like tumor–neuron interactions. Scale bar, 50 μm. Data are presented as mean ± SEM. Statistical significance was determined using one-way ANOVA followed by Tukey’s post hoc test

To determine whether anti-VEGF treatment is associated with acquisition of synaptic scaffold-marker features in GBM cells, we established primary cultures from vehicle-derived and anti-VEGF–treated IRP orthotopic GBM tumors and co-cultured them with mouse cortical neurons. Cultures were stained for Nestin to define GSCs regions and for postsynaptic scaffold markers SAP97/DLG1 and PSD95/DLG4. Compared with vehicle-derived cultures, anti-VEGF–treated IRP-derived cultures showed increased SAP97/DLG1-positive puncta and PSD95/DLG4-positive signal within Nestin-positive GBM-cell regions (Fig. 5a,b and S3a).

We next examined whether this postsynaptic-associated phenotype was linked to the CTNNB1–NRG3–ERBB4 signaling axis. CTNNB1-Δ90 expression increased SAP97/PSD95-positive puncta/signals in GBM cells maintained in neuron-containing co-culture, whereas siRNA-mediated silencing of NRG3 or ERBB4 reduced this punctate phenotype within Nestin-positive GBM-cell regions. Quantitative analysis within Nestin-positive GBM-cell regions confirmed that anti-VEGF–treated IRP-derived cultures and CTNNB1 activation increased postsynaptic scaffold-marker positivity, while NRG3 or ERBB4 silencing suppressed this response. Together, these findings indicate that anti-VEGF resistance is accompanied by a postsynaptic scaffold-marker phenotype in GBM cells and suggest that CTNNB1 promotes this phenotype through downstream NRG3–ERBB4 signaling.

To further define the β-catenin activation state associated with the IRP-linked postsynaptic program, we analyzed β-catenin phosphorylation and nuclear localization in IRP- and LRP-derived GBM cell lines. We focused on phosphorylation sites with distinct functional consequences: N-terminal phosphorylation at Ser33/Ser37/Thr41 is associated with β-catenin degradation, whereas Ser675 phosphorylation is associated with β-catenin transcriptional activation [45, 53]. Immunoblot analysis showed that IRP-associated GBM cells displayed increased activation-linked β-catenin signaling, including elevated p-β-catenin Ser675 and increased nuclear β-catenin, together with higher expression of ERBB4, MAP2, and PSD95 under WNT3A-stimulated conditions with enhanced nuclear β-catenin accumulation. In contrast, treatment with PRI-724, a β-catenin/CBP-dependent transcriptional inhibitor, reduced nuclear β-catenin-associated signaling and decreased ERBB4, MAP2, and PSD95 expression. Conversely, WNT3A stimulation enhanced β-catenin pathway activation and increased these neuronal/postsynaptic-associated markers (Fig. 5c and S3b). These results indicate that β-catenin transcriptional activity is coupled to ERBB4 expression, MAP2-associated neuronal remodeling, and PSD95/DLG4 and SAP97/DLG1-associated postsynaptic signaling, supporting a mechanistic link between WNT/β-catenin activation and the postsynaptic-like response phenotype observed during the IRP.

To determine whether the response phenotype of IRP-derived GBM cells depends on PSD95-associated postsynaptic interactions, we compared control and siPSD95-treated IRP cell lines under therapy pressure though PRI-724 treatment. Control IRP cells maintained limited expansion and survival, consistent with their resistant phenotype, whereas siRNA-mediated PSD95/DLG4 silencing converted these cells toward a therapy-sensitive state under the treatment, markedly reducing their growth/survival advantage (Fig. 5d and S3c). These findings indicate that PSD95/DLG4-associated postsynaptic signaling is functionally required for maintaining IRP-associated response, rather than serving merely as a marker of neuronal-like remodeling. Consistent with this, immunofluorescence analysis showed that control IRP cells formed abundant PSD95/DLG4-positive postsynaptic-associated signals in co-culture with neurons. In contrast, PSD95/DLG4 silencing reduced these signals and weakened synapse-associated tumor–neuron interactions (Fig. 5e). Quantification of discrete signals confirmed that loss of PSD95/DLG4 diminished the postsynaptic-associated interactions.

Together, these results support a model in which anti-VEGF therapy induces a response-associated postsynaptic phenotype in GBM cells. Mechanistically, CTNNB1 activation promotes NRG3–ERBB4-dependent postsynaptic-like tumor–neuron interactions, linking neuronal-like remodeling to survival advantage under therapeutic stress.

### Combined anti-VEGF and β-catenin/CBP inhibition attenuates adaptive neuronal programs and prolongs survival in orthotopic GBM PDX models

To determine the therapeutic relevance of the CTNNB1–NRG3–ERBB4 signaling axis in GBM adaptation to anti-VEGF therapy, we pharmacologically targeted the β-catenin using PRI-724, a clinically evaluated β-catenin/CBP interaction inhibitor (NCT01606579). Because our mechanistic data indicated that β-catenin activity promotes NRG3–ERBB4-associated neuronal and postsynaptic-like adaptive programs, we reasoned that inhibition of β-catenin/CBP-dependent signaling axis could attenuate this resistance-associated state (Fig. S4a).

**Fig. 6:**
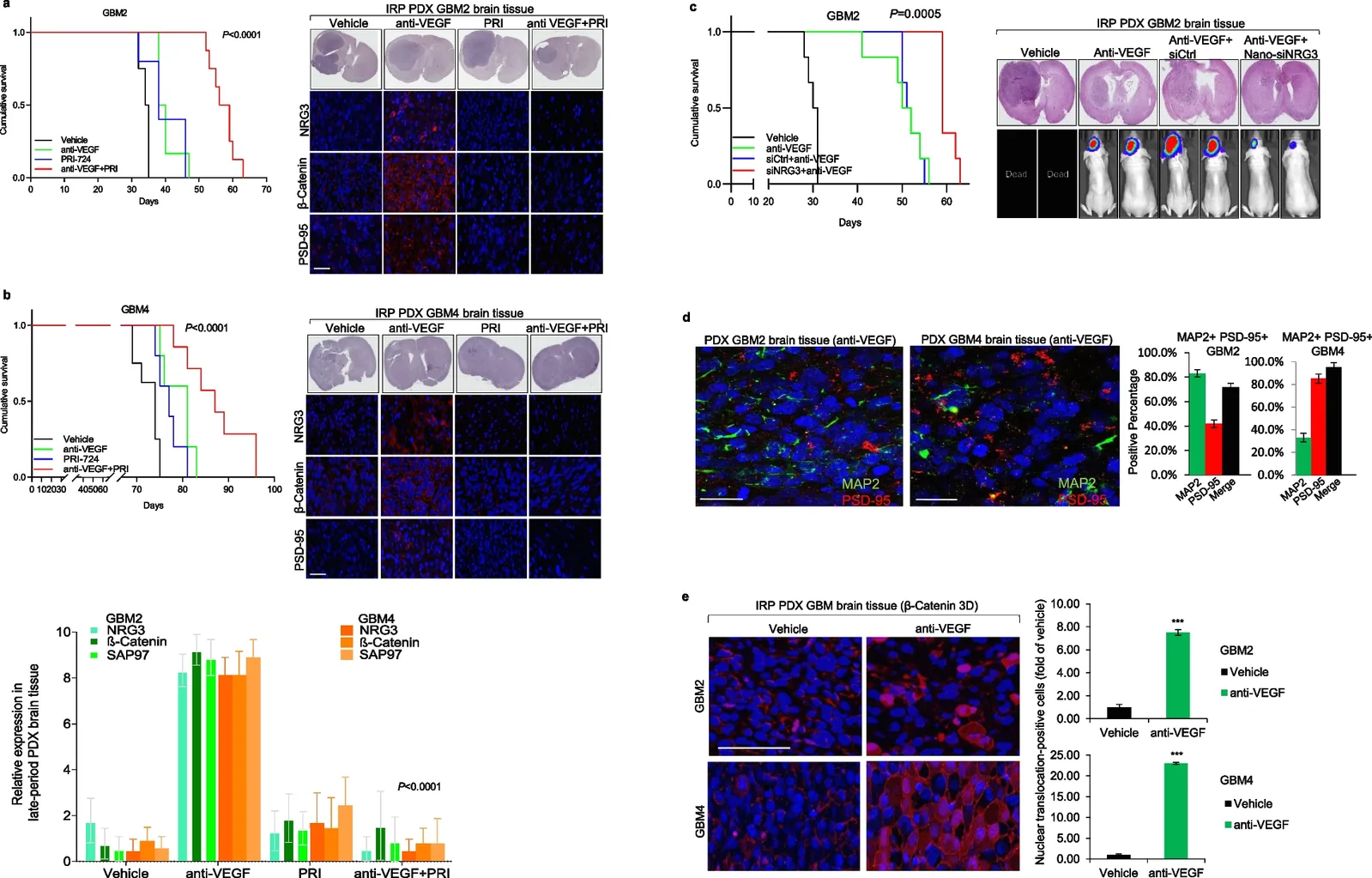
Combination of VEGF-targeted therapy with PRI-724 suppresses neuronal-like activity transition and increases survival in orthotopic PDX mouse models. **a**,**b**. Kaplan–Meier survival analysis of orthotopic GBM2 and GBM4 PDX models treated with vehicle, anti-VEGF therapy, PRI-724, or combined anti-VEGF + PRI-724 treatment. Upper, survival analysis of mice implanted with GBM2 cells; mice received vehicle, anti-VEGF therapy, PRI-724, or combination treatment (*n* = 8, 8, 8, and 8 mice per group, respectively; 1 × 10^4^ cells implanted per mouse). Median survival times were 34.5, 39, 38, and 57.5 days, respectively. Lower, survival analysis of mice implanted with GBM4 cells; mice received vehicle, anti-VEGF therapy, PRI-724, or combination treatment (*n* = 8, 8, 8, and 8 mice per group, respectively; 1 × 10^5^ cells implanted per mouse). Median survival times were 74, 77, 81, and 88 days, respectively. Survival differences were assessed using the log-rank (Mantel–Cox) test, with pairwise comparisons performed between the combination group and each monotherapy group. Representative immunofluorescence images and quantification of NRG3, β-catenin, and PSD95/DLG4 in GBM2 and GBM4 orthotopic PDX tumors collected at the predefined fourth imaging time point during the IRP after vehicle, anti-VEGF, PRI-724, or combined anti-VEGF + PRI-724 treatment. Combination treatment reduced NRG3, β-catenin, and PSD95/DLG4 signals, indicating suppression of the NRG3–β-catenin-associated postsynaptic adaptive program during the IRP. Quantification was performed from three independent experiments. Data are presented as mean ± SEM; one-way ANOVA with Tukey’s post hoc test. Scale bar, 50 μm. Anti-VEGF + PRI-724 vs vehicle *p* = 0.0001 (a,b) Anti-VEGF + PRI-724 vs anti-VEGF *p* = 0.0001 (a,b), Anti-VEGF + PRI-724 vs PRI-724 *p* = 0.0001 (a,b), Anti-VEGF vs vehicle *p* = 0.001 (a,b) PRI-724 vs vehicle *p* = 0.001 (a,b), **c** Kaplan–Meier survival analysis and representative tumor-burden assessment in orthotopic GBM2 PDX mice treated with vehicle, anti-VEGF therapy, Nano-siCtrl, or Nano-siNRG3 + anti-VEGF therapy. GBM2-luc cells were orthotopically implanted at 3 × 10^5^ cells per mouse, and mice were assigned to each treatment group (*n* = 6 per group). Median survival times were 30.5, 51, 51.5, and 59 days for vehicle, anti-VEGF therapy, Nano-siCtrl, and Nano-siNRG3 groups, respectively. Survival differences were assessed using the log-rank (Mantel–Cox) test, with pairwise comparisons performed between the Nano-siNRG3 group and the indicated each monotherapy group. Representative H&E-stained brain sections and BLI images show reduced tumor burden in Nano-siNRG3-treated mice compared with control groups. “Dead” indicates animals that reached predefined humane endpoint criteria before the corresponding imaging time point. Data are presented as mean ± SEM; one-way ANOVA with Tukey’s post hoc test. Scale bar, 50 μm. Anti-VEGF + Nano-siNRG3 vs vehicle *p* = 0.0001, Anti-VEGF + Nano-siNRG3 vs anti-VEGF *p* = 0.0005, Anti-VEGF + Nano-siNRG3 vs Nano-siNRG3 p = 0.0005, Anti-VEGF vs vehicle p = 0.001, Nano-siNRG3 vs vehicle *p* = 0.001 **d** Representative immunofluorescence images of MAP2 and PSD95/DLG4 co-staining in GBM2,4 orthotopic PDX brain tumor tissue (at IRP). MAP2 and PSD95/DLG4 were used to visualize neuronal- and postsynaptic scaffold-associated marker expression, respectively. This panel provides complementary spatial validation of the neuronal/postsynaptic-associated signature observed in the proteomic and immunoblot analyses. Nuclei were counterstained with DAPI. Scale bar, 50 μm. The positive co-staining expression was quantified in three biologically independent tumors per group. Data are presented as mean ± SEM. Statistical significance was determined using one-way ANOVA followed by Tukey’s post hoc test. **e** Representative 3D confocal images of GBM4 orthotopic PDX brain tumor tissue from Fig. B showing β-catenin localization. β-catenin showed nuclear accumulation within tumor regions, consistent with WNT/β-catenin pathway activation. Nuclei were counterstained with DAPI. Scale bar, 50 μm. Nuclear β-catenin was quantified in three biologically independent tumors per group. Data are presented as mean ± SEM. Statistical significance was determined using one-way ANOVA followed by Tukey’s post hoc test

To assess the in vivo therapeutic efficacy of β-catenin/CBP inhibition in combination with anti-VEGF therapy, we used orthotopic PDX models derived from GBM2 and GBM4. GBM2 was selected as a representative mechanistic model because it displayed robust IRP-associated WNT/β-catenin activation and neuronal/postsynaptic adaptive programs under anti-VEGF therapy. GBM4 was included as an independent responder-derived validation model that preserved anti-VEGF response-associated neuronal and synapse-related features in patient-derived analyses. Compared with anti-VEGF therapy or PRI-724 monotherapy, the combination regimen significantly prolonged survival by approximately 20–30% in tumor-bearing mice (Fig. 6a,b).

Consistent with the survival benefit, immunofluorescence analysis showed that combined anti-VEGF and PRI-724 treatment reduced resistance-associated WNT/neuronal and postsynaptic markers in orthotopic GBM PDX tumors. In anti-VEGF–treated tumors, NRG3, β-catenin, and PSD95/DLG4 signals were readily detected within tumor regions, consistent with activation of the CTNNB1–NRG3–ERBB4-associated neuronal/postsynaptic adaptive program. In contrast, combination treatment markedly reduced NRG3, β-catenin, and PSD95/DLG4 expression (Fig. 6a,b). These tumors were collected at the fourth (4th) during the IRP, corresponding to a transition window in which WNT/neuronal-associated and postsynaptic-like adaptive features are prominent in our longitudinal PDX model. The reduction of NRG3, β-catenin, and PSD95/DLG4 after combination treatment suggests that β-catenin/CBP inhibition attenuates the NRG3-associated postsynaptic adaptive state induced during anti-VEGF therapy.

To determine whether NRG3 represents an actionable extracellular component of the adaptive neuronal program, we used nanoparticle-mediated siRNA delivery in orthotopic GBM PDX models. Nano-siNRG3 treatment reduced tumor burden, as assessed by BLI and histological analysis, and prolonged survival compared with vehicle, anti-VEGF therapy alone, or nanoparticle-delivered control siRNA (Nano-siCtrl) (Fig. 6c). These findings provide in vivo proof-of-concept that NRG3 silencing can impair the neuronal adaptive program and delay tumor progression under anti-VEGF therapy pressure.

In addition, complementary immunofluorescence analysis of anti-VEGF–treated GBM PDX tissue showed MAP2 and PSD95/DLG4 co-staining within the same tumor region, supporting the presence of neuronal/postsynaptic-associated marker expression in vivo. Consistent with activation of the upstream WNT/β-catenin component of this adaptive program, representative 3D confocal imaging of anti-VEGF–treated GBM PDX brain tumor tissue showed β-catenin nuclear accumulation within tumor regions (Fig. 6d,e).

Together, these results support a mechanistically guided therapeutic framework in which β-catenin/CBP inhibition and NRG3-directed silencing target complementary components of anti-VEGF-associated adaptive resistance. By suppressing β-catenin-dependent adaptation and extracellular NRG3 signaling, these interventions attenuate neuronal/postsynaptic adaptive states and delay tumor escape from anti-VEGF therapy in orthotopic GBM models.

### Patient-derived analyses support neuronal and synaptic adaptive remodeling associated with anti-VEGF response in GBM

**Fig. 7:**
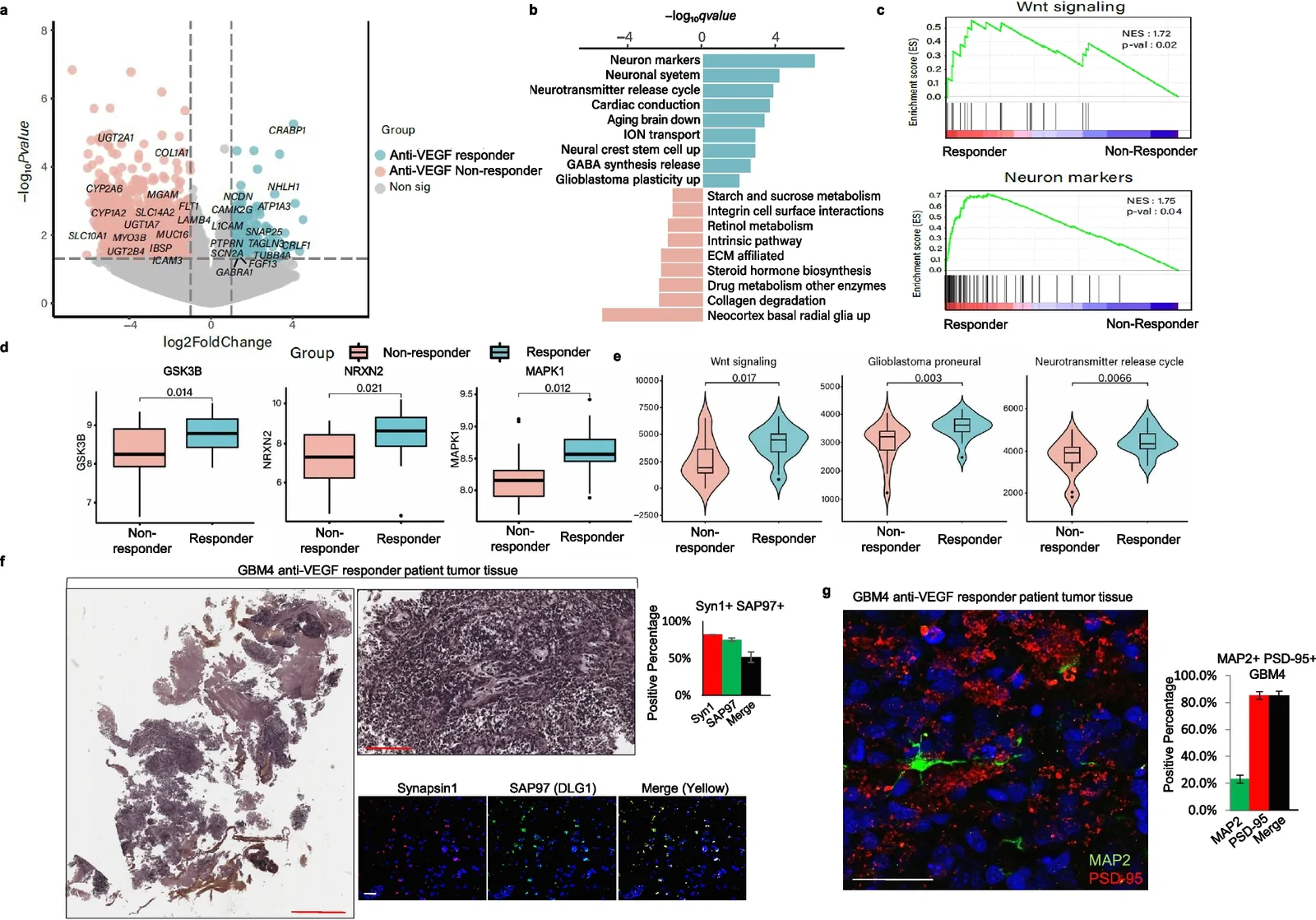
Patient-derived analyses support neuronal and postsynaptic-like adaptive remodeling associated with anti-VEGF response in GBM. **a** Volcano plot showing differential mRNA expression between anti-VEGF responder and non-responder tumors from the GSE79671 patient dataset. Responders and non-responders were defined according to clinical response to anti-VEGF therapy. Neuronal differentiation-, neurodevelopmental-, and synapse-associated genes, including CAMK2G, L1CAM, GABRA1, FGF13, SNAP25, SCN2A, ATP1A3, NCDN, NHLH1, and PTPRN, were enriched in responder-associated tumors. Genes are plotted by log2 fold change and − log10 nominal P value. Nominal P values are shown for visualization, whereas statistical significance was defined using Benjamini–Hochberg FDR-adjusted q values. **b** Pathway enrichment analysis of genes differentially enriched in anti-VEGF responder and non-responder tumors. Responder-associated tumors showed enrichment of neuronal and synapse-related programs, whereas non-responder tumors were associated with drug metabolism- and extracellular matrix-related programs. Bar length represents − log10 FDR-adjusted q value. **c** Gene set enrichment analysis showing enrichment of WNT signaling and neuronal marker programs in anti-VEGF responder tumors compared with non-responder tumors. Normalized enrichment scores (NES) and nominal P values are indicated in each plot. GSEA-derived FDR q values were used to assess multiple testing across gene sets, with the significance threshold indicated in the Methods. **d** Box plots showing mRNA expression of GSK3B, NRXN2, and MAPK1 in anti-VEGF responder and non-responder tumors. GSK3B is shown as a WNT pathway-associated transcript and is interpreted cautiously as a potential feedback-associated component of WNT pathway remodeling, rather than as direct evidence of β-catenin nuclear translocation. NRXN2 and MAPK1 represent neuronal/synapse-associated signaling regulators. Group differences were assessed using the Wilcoxon rank-sum test, and values above brackets indicate nominal P values. Boxes indicate the interquartile range, center lines indicate the median, and whiskers indicate the data range unless otherwise indicated. **e** Violin plots showing pathway-level activity scores for WNT signaling, glioblastoma proneural programs, and neurotransmitter release pathways in anti-VEGF responder and non-responder tumors. Responder tumors showed increased activity of WNT-associated neuronal and synapse-related programs. Pathway activity scores were compared between groups using the Wilcoxon rank-sum test, and values above brackets indicate nominal *P* values. Multiple pathway testing was controlled using Benjamini–Hochberg FDR correction where applicable. **f** Histological and immunofluorescence analysis of GBM4 patient tumor tissue from a clinically defined anti-VEGF responder. Left, low-magnification H&E image of surgically resected GBM4 patient tumor tissue. Middle, higher-magnification H&E image showing tumor-enriched regions used for marker analysis. Lower panels show representative immunofluorescence images stained for SAP97/DLG1, and SYN1, with merged images shown to visualize spatial overlap between synapse-associated marker signals. SAP97/DLG1 was assessed as a postsynaptic scaffold marker, and SYN1 was assessed as a synapse-associated marker. Quantification shows the percentage of SAP97/DLG1- or SYN1-positive signal, and overlapping signals. Scale bars: 4 mm for low-magnification H&E, 300 μm for high-magnification H&E, and 50 μm for immunofluorescence images. Data are presented as mean ± SEM. **g** Representative immunofluorescence image of MAP2 and PSD95/DLG4 co-staining in GBM4 patient tumor tissue from a clinically defined anti-VEGF responder. MAP2 was assessed as a neuronal/dendritic-associated marker, and PSD95/DLG4 was assessed as a postsynaptic scaffold marker. The merged image shows spatial proximity and partial overlap of MAP2- and PSD95/DLG4-positive signals within the same tumor region, supporting the presence of neuronal/postsynaptic-associated features in responder tumor tissue. Scale bar, 50 μm

To evaluate the clinical relevance of neuronal and postsynaptic adaptive remodeling identified in our longitudinal PDX models, we analyzed the available dataset GSE79671 from GBM patients treated with anti-VEGF therapy [54]. This clinically annotated paired dataset includes tumor samples collected before anti-VEGF treatment and at progression after treatment, allowing us to assess whether anti-VEGF-responsive tumors acquire treatment-associated adaptive programs resembling the neuronal and synaptic states identified in our PDX models. Differential expression analysis of paired samples from responding patients revealed treatment-associated enrichment of neuronal differentiation-, neurodevelopmental-, and synapse-associated genes, including CAMK2G, L1CAM, GABRA1, FGF13, SNAP25, SCN2A, ATP1A3, NCDN, NHLH1, and PTPRN [55] (Fig. 7a,b). These genes are linked to calcium-dependent neuronal signaling, neural cell adhesion and axonal guidance, GABAergic neurotransmission, neuronal excitability, ion homeostasis, neurodevelopmental transcriptional regulation, and synaptic vesicle-associated function. Together, these findings indicate that anti-VEGF-responsive GBM tumors acquire treatment-associated neuronal and synapse-associated adaptive programs, consistent with the neuronal and postsynaptic states identified in our longitudinal PDX models.

Gene set enrichment analysis (GSEA) further demonstrated that anti-VEGF-responsive tumors were enriched for WNT signaling and neuronal gene programs compared with non-responsive tumors (Fig. 7c). GSK3B was also elevated among WNT-associated transcripts, potentially reflecting feedback-associated WNT pathway remodeling. In parallel, neuronal and synapse-associated regulators, including NRXN2, a synaptic adhesion molecule linked to neuronal connectivity and proneural GBM programs, and MAPK1, a signaling kinase involved in neuronal survival and plasticity, were also increased [56] (Fig. 7d). Together, these data indicate that anti-VEGF-responsive GBM tumors exhibit WNT-associated neuronal and synapse-related adaptive programs, consistent with the neuronal/postsynaptic states identified in our longitudinal PDX models.

Pathway-level analyses demonstrated that anti-VEGF-responsive tumors exhibited enhanced WNT signaling, proneural transcriptional programs, and neurotransmitter release pathways (Fig. 7e). In contrast, non-responsive tumors were associated with drug metabolism- and extracellular matrix-related programs in the differential expression analysis. These data suggest that anti-VEGF response is accompanied by distinct adaptive molecular programs, with responsive tumors showing enrichment of neuronal and synapse-associated states.

To determine whether these patient-derived signatures were reflected at the protein level, we performed immunofluorescence analysis using our GBM responder-derived patient tumor tissue. GBM4 patient tumor tissue from a clinically defined anti-VEGF responder displayed synapse-associated and postsynaptic scaffold-marker expression, including SYN1 as a synapse-associated marker and SAP97/DLG1 as postsynaptic scaffold markers. Complementary MAP2 and PSD95/DLG4 co-staining further supported the presence of neuronal/postsynaptic-associated marker expression in responder tumor tissue (Fig. 7f,g). These patient-derived protein-level observations were concordant with the molecular features identified in our experimental models, while their broader prevalence and association with treatment response remain to be evaluated in larger clinically annotated cohorts.

Overall, these patient analyses and GBM4-derived protein-level result support the clinical relevance of WNT-associated neuronal and postsynaptic adaptive programs identified in our longitudinal PDX models.

## Discussion

In this study, we delineated a temporally organized and longitudinally traceable trajectory of adaptive resistance in GBM, identifying CTNNB1–NRG3–ERBB4-driven neuronal/postsynaptic-like remodeling as an initial adaptive program during anti-VEGF therapy. Despite major advances in multimodal treatment, GBM remains therapeutically unresolved. anti-VEGF therapy has been used for primary or recurrent GBM, often in combination with temozolomide, irinotecan, or lomustine. Although these approaches can transiently improve PFS, they have not consistently produced durable gains in OS, underscoring the need to define the mechanisms by which GBM adapts during therapy [4, 5, 57–61]. Our longitudinal orthotopic PDX strategy enabled time-resolved tracking of tumor adaptation the IRP and the LRP, revealing that anti-VEGF resistance is not a static endpoint but a staged adaptive process involving tumor-intrinsic signaling, postsynaptic remodeling, clonal dynamics, and microenvironmental reorganization.

A central finding of this study is that the IRP is not simply a period of passive tumor suppression. Rather, GBM cells surviving early anti-VEGF pressure activate a WNT/β-catenin-associated neuronal and postsynaptic adaptive state. This state was most apparent at the protein and phosphoprotein levels, highlighting the value of proteomic profiling for detecting rapid functional adaptation that may not be fully captured by transcript or mutation abundance alone.

Mechanistically, our data support a signaling hierarchy in which β-catenin-dependent transcription engages NRG3–ERBB4 signaling to promote neuronal-like remodeling. CTNNB1 activation increased NRG3-associated signaling, whereas disruption of NRG3 or ERBB4 attenuated downstream neuronal marker induction. These findings place NRG3–ERBB4 signaling downstream of β-catenin-associated adaptation and upstream of postsynaptic remodeling. A key advance of this work is the functional validation of the postsynaptic-like markers. PSD95/DLG4 silencing reduced postsynaptic-associated puncta and attenuated the resistance-associated growth and survival advantage of IRP-derived GBM cells. Thus, postsynaptic-like remodeling is not merely a marker of neuronal-like remodeling, but a functional component of the adaptive resistance phenotype..

These findings are consistent with and extend a growing body of work showing that GBM cells exploit neuronal activity and synaptic signaling to support tumor growth and adaptive plasticity. Recent studies have highlighted NLGN3-mediated paracrine signaling, BDNF–TrkB-driven malignant synaptic plasticity, AMPA receptor-mediated neuron-to-glioma synapses, and GABAergic neuron-to-glioma synaptic mechanisms [62–67], underscoring the biological relevance of neuronal communication in glioma progression. Our study extends this framework by defining an anti-VEGF-associated temporal adaptive trajectory and identifying the CTNNB1–NRG3–ERBB4–PSD95/SAP97 signaling program as a functionally validated component of neuronal remodeling in therapy-adapted GBM cells. Importantly, we interpret these findings as evidence of therapy-induced neuronal scaffold-marker-positive remodeling rather than definitive mature functional synapse formation.

Patient-derived analyses further supported the clinical relevance of the neuronal and synapse-associated programs identified in our PDX models. Because sequential sampling of IRP or LRP is rarely feasible in patients, we used the clinically annotated GSE79671 dataset as supportive evidence for anti-VEGF-associated adaptive programs in GBM. Analysis of paired pre- and post-treatment tumors revealed enrichment of neuronal differentiation-, neurodevelopmental-, and synapse-associated programs. In addition, GBM4 responder-derived patient tissue (n = 1) showed increased synapse-associated and neuronal-associated proteins, including PSD95, SAP97, MAP2, and SYN1. These patient-derived observations were concordant with the molecular features identified in our experimental models, while their broader prevalence and association with treatment response remain to be evaluated in larger clinically annotated cohorts. Larger clinically annotated cohorts incorporating matched primary/recurrent specimens, treatment-response information, molecular profiling, and survival outcomes will be required to determine whether the CTNNB1–NRG3–ERBB4-associated neuronal-like remodeling predicts clinical outcome or therapeutic response [68, 69].

Therapeutically, our findings suggest that anti-VEGF adaptive resistance can be targeted at multiple complementary levels. PRI-724, a clinically evaluated investigational β-catenin/CBP interaction inhibitor, enhanced the therapeutic effect of anti-VEGF therapy and prolonged survival in orthotopic GBM PDX models. Although prior human studies have evaluated PRI-724-related β-catenin/CBP inhibition for safety and tolerability, the optimal dose, route, schedule, and combination strategy for GBM remain to be defined. This supports the concept that the β-catenin arm of the CTNNB1–NRG3–ERBB4 axis is therapeutically targetable. In parallel, nanoparticle-mediated NRG3 silencing reduced tumor burden and prolonged survival, providing in vivo proof-of-concept that the extracellular NRG3 component contributes to the adaptive neuronal program.

Several limitations should be considered. First, the primary PDX models used to define the IRP/LRP trajectory were IDH1/2-wild-type and MGMT promoter-unmethylated, and therefore represent a clinically GBM subgroup rather than the full molecular diversity of GBM. The present findings were derived from IDH-wildtype glioblastoma models and should not be directly extrapolated to astrocytoma, IDH-mutant, CNS WHO grade 4, which represents a distinct molecular and diagnostic entity [43]. Further studies are required to determine whether similar anti-VEGF-induced neuronal and postsynaptic adaptive programs occur in IDH-mutant astrocytoma. Second, although longitudinal MRI enabled tumor-volume tracking in orthotopic models, imaging-based phase definition has important limitations. Not all GBM patients exhibit a clearly biphasic radiographic response pattern equivalent to the experimentally defined IRP and LRP; however, a subset of patients may show initial radiographic stabilization or slower progression followed by accelerated regrowth. In clinical and preclinical settings, T2-Weighted-based tumor measurements can be confounded by edema, treatment-induced vascular normalization, and changes in blood–brain barrier (BBB) permeability, and discrepancies may arise between weighted and gadolinium-enhanced MRI measurements. To mitigate this limitation, we complemented MRI with BLI to monitor viable tumor burden independent of contrast enhancement. However, because BLI is not clinically applicable and murine BBB physiology differs from that of patients, clinical translation of this framework will require integration of gadolinium-enhanced MRI, weighted imaging, clinical response features, and molecular or immunohistopathological biomarkers to identify patients most likely to benefit from therapies targeting the CTNNB1–NRG3–ERBB4 axis. Third, patients in the GSE79671 cohort received bevacizumab in combination with irinotecan rather than bevacizumab monotherapy. Although irinotecan is not known to selectively induce neuronal or synapse-associated transcriptional programs, its independent or interactive contribution to the observed changes cannot be excluded. Fourth, VEGF blockade can reduce vascular permeability, edema, and contrast enhancement without producing a proportional reduction in viable tumor burden, resulting in radiographic pseudoresponse. Although tumor burden in this study was evaluated using serial MRI measurements rather than contrast enhancement alone, these measurements may still be influenced by treatment-associated changes in vascular permeability and tissue water content. Therefore, the MRI-defined IRP should be interpreted as an operational imaging-associated state of tumor stabilization or reduced apparent growth rather than definitive tumor regression.

In summary, our study reframes anti-VEGF resistance in GBM as a temporally ordered adaptive process involving early CTNNB1–NRG3–ERBB4-driven neuronal-like remodeling followed by broader ECM, mesenchymal. By integrating longitudinal orthotopic PDX models, proteomic and phosphoproteomic profiling, patient-derived validation, and functional intervention, we identify neuronal/postsynaptic adaptation as a targetable vulnerability during early therapy response. These findings provide a rationale for phase-informed combination strategies designed to suppress adaptive neuronal states and delay tumor escape from anti-VEGF therapy.

## Materials and methods

### Patient-derived glioblastoma specimens and derived cell cultures

For all human studies, informed consent was obtained and tissue was used in accordance with protocols approved by the National Cancer Center Institutional Review Board (IRB). pri-GBM1, pri-GBM2, pri-GBM3, and pri-GBM4 were obtained as described in our previous reports or newly isolated using Patel’s method (RNA-seq based classification and treatment history, grade IV, IDH1-wild type and MGMT-unmethylated) [8–17]. For all patient-derived cultures, mycoplasma testing was routinely performed every 6 months and before major in vitro and in vivo experiments. Short tandem repeat (STR) DNA profiling was used to verify culture identity by comparison with the corresponding parental GBM1–4 tumor tissue profiles obtained from the National Cancer Center. Cultures were considered authenticated when their STR profiles were concordant with those of the matched parental tumor specimens at the evaluable STR loci. All patient-derived cultures were obtained from the National Cancer Center and were additionally characterized using RNA-sequencing data. In brief, tissues of GBM4 were obtained from a high-grade glioma (WHO (World Health Organization) grade IV, IDH1-wild type and MGMT no methylation) tumor at the time of biopsy. The GBM4 responders sample for MRI and immunofluorescence analysis was from the occipital at the time of biopsy from a male who was 45 years of age at diagnosis in the National Cancer Center Institution, treated with surgical resection, radiotherapy, temozolomide, and bevacizumab treatment. The GBM4 responder samples were evaluated based on the RANO criteria. Responders were defined as patients with complete or partial response (CR + PR).

### Animal experiments and orthotopic xenografts

All animal procedures were approved by the Institutional Animal Care and Use Committee of the National Cancer Center Research Institute (NCCRI; protocol no. NCC-17–402). NCCRI is accredited by AAALAC International, and animal experiments were performed in accordance with the ILAR Guide for the Care and Use of Laboratory Animals. For intracranial implantation, mice were anesthetized with Zoletil 50 (Carros, France). GSCs were resuspended in either DMEM or DMEM/F-12 medium supplemented with B27, EGF (10 ng/mL), and bFGF (5 ng/mL), according to the respective experimental condition. Cell suspensions were stereotactically implanted into the left striatum of 5-week-old BALB/c nude mice. The injection site was positioned 2.2 mm lateral to the midline and 0.2 mm posterior to bregma, at a depth of 3.5 mm. Both sexes were included and allocated to experimental groups without sex-based stratification. The study was not designed or powered to assess sex-specific effects.

### MRI Images

MRI was performed weekly using a Bruker BioSpec 7 T system (BioSpec 70/20 USR; Bruker, Germany) equipped with a mouse brain array coil. Mice were anesthetized with isoflurane (1–1.5%), and respiration was monitored throughout the scan. T2-weighted images were acquired using a RARE sequence (TR/TE, 2500/35 ms; slice thickness, 0.7 mm; RARE factor, 8; NEX, 4; matrix, 256 × 192; FOV, 20 × 20 mm) and processed with ParaVision 5.1 (Bruker, Germany). Pre-sacrifice MRI was used to localize intracranial tumors and facilitate anatomical matching with brain sections. Coronal sections were aligned with the corresponding MRI planes using anatomical landmarks, and adjacent H&E-stained sections were examined to identify viable tumor regions for macrodissection. Regions containing ≥ 90 ± 5% tumor cells were selected for molecular analyses. Tumor dimensions were measured in the three orthogonal planes: craniocaudal diameter (dcc) on sagittal images, anteroposterior diameter (dap) on coronal images, and maximum lateral diameter (dl) on axial images. Tumor volume was estimated assuming an ellipsoidal shape using V = dcc × dap × dl × π/6.

### Transfection

siRNAs were transfected, briefly, 3 × 10^5^ GBM cells were plated on 60-mm culture dishes, incubated for 24 h, and then transfected with 20 pmol of siRNA constructs, using 15 μl of Lipofectamine 2000 (Invitrogen). The GBM cells were harvested 72 h, 120 h (coculture) after transfection. siNRG3 #1, sense 5′- AGAAGAUGUGGGCAAUUCAUU-3′ and antisense 5′-UGAAUUGCCCACAUCUUCUUU-3′; siNRG3 #2, sense 5′-GCUGUAAGCACAUGUGUUCUU-3′ and antisense 5′-GAACACAUGUGCUUACAGCUU-3′; siNRG3 #3, sense 5′-GUUCAUUGUGCGUAUGUGUUU-3′ and antisense 5′-ACACAUACGCACAAUGAACUU-3′; siNRG3 Negative control, sense 5′-CCUCGUGCCGUUCCAUCAGGUAGUU-3′ and antisense 5′-CUACCUGAUGGAACGGCACGAGGUU-3′; siERBB4 #1, sense 5′-CGUGUUAGUGGCUCUUAAUUU-3′ and antisense 5′-AUUAAGAGCCACUAACACGUU-3′; siERBB4 #2, sense 5′-CCAAGAUGGAAGUAGAAGAUU-3′ and antisense 5′-UCUUCUACUUCCAUCUUGGUU-3′; siERBB4 #3, sense 5′- CCAUAGACCCAGAGAAACUUU-3′ and antisense 5′-AGUUUCUCUGGGUCUAUGGUU-3′; siERBB4 Negative control, sense 5′-CCUCGUGCCGUUCCAUCAGGUAGUU-3′ and antisense 5′-CUACCUGAUGGAACGGCACGAGGUU-3′. DLG4, sense 5′-GCAUGAGUGGAAGGUCUAAAU-3′ and antisense 5′-AUUUAGACCUUCCACUCAUGC-3′

### Immunoblot analysis

Proteins were extracted with RIPA buffer with complete protease inhibitors (Roche), separated by electrophoresis, transferred to PVDF membranes (Millipore), and blocked with 5% skim milk (BD). Primary antibodies against MAP2 (SYSY, 188 003, 1:1000, 75 kDa), ERBB4 (Abcam, ab219208, 1:1000, 180 kDa), NRG3 (Invitrogen, PA5-42,602, 1:1000, 80 kDa), PSD95 (NOVUS, NBP1-80,875, 1:1000, 95 kDa), SAP97(GeneTex, GTX64496, 1:300), Myc (C47E12, Cell Signaling Technology, #3395, 1:1000, 200 kDa), p-β-catenin S33/37/41 (Cell Signaling Technology, #9561, 1:1000, 92 kDa), p-β-catenin S675 (Cell Signaling Technology, #9567, 1:1000, 92 kDa), β-catenin (Cell Signaling Technology, #8480, 1:1000, 92 kDa), and β-tubulin (Santa Cruz Biotech, sc-47778, 1:1000, 43 kDa) were incubated overnight at 4 °C. Immunoreactive bands were visualized using peroxidase-labeled affinity-purified secondary antibodies (KPL) and the Amersham ECL Prime Western blotting detection reagent (GE Healthcare). Patient-derived GBM1, GBM2, and GBM3 cells were maintained under the established culture conditions described above. Unless otherwise indicated, IRP-derived cells were generated from anti-VEGF–treated orthotopic PDX tumors collected at the fourth treatment time point, whereas LRP-derived cells were generated from tumors collected at the fifth treatment time point. For pathway modulation, cells were treated with PRI-724 at 300 ng/mL for 48 h or recombinant WNT3A at 100 ng/mL for 48 h before protein extraction. Cells were lysed in protein lysis buffer, and protein concentrations were measured by BCA assay. Equal amounts of protein, typically 20 μg per lane.

### Immunohistochemistry and histology

For histological and immunostaining analyses, brains were harvested, fixed in 4% PFA for 24 h at 4 °C, and sectioned at 4 μm using a Leica RM2125 RTS microtome. Tissue sections were deparaffinized, rehydrated, and subjected to citrate-based antigen retrieval (pH 6.0). After washing, sections were permeabilized with 0.1% Triton X and blocked for 24 h at room temperature. H&E staining was performed using hematoxylin (Dako) and 0.25% eosin (Merck). For immunohistochemistry and immunofluorescence, sections were incubated overnight at 4 °C with primary antibodies against Nestin (Abcam, ab6320, 1:300) HIF-1a (BETHYL, BL-124-3F7, 1:300), PSD95 (NOVUS, NBP1-80,875, 1:300), SAP97(GeneTex, GTX64496, 1:300), Periostin (Abcam, ab219056, 1:100), GRIP2 (Abcam, ab171411, 1:300), FGFR1 (Invitrogen, PA1-24,762, 1:300), β-catenin (Cell Signaling Technology, #8480, 1:300), CD44 (Abcam, ab51037, 1:300), TGM2 (Cell signaling, #3557, 1:300), parvalbumin (SWANT, PV 27, 1:300), Pre-proenkephaline (NEUROMICS, RA14124, 1:300). NeuN (Millipore, MAB377, 1:300), MAP2 (SYSY, 188 003, 1:300), Synapsin1 (SYSY, 106 011, 1:300), cleaved-Caspase3 (Cell Signaling, #9661, 1:300) and Tuji1 (Lsbio, LS-B225-75, 1:300). For chromogenic immunohistochemistry, endogenous peroxidase activity was quenched with 3% hydrogen peroxide. For immunofluorescence, appropriate Alexa Fluor 488- or 568-conjugated secondary antibodies (Thermo Fisher Scientific) were applied following primary antibody incubation.

### Ang-SS-siRNA nanocapsule treatment of a brain tumor mouse orthotopic xenograft model.

Small interfering RNAs (siRNAs) were synthesized by GenePharma Co. Ltd. (Shanghai, China). siRNA for human NRG3 (5′- GUUCAUUGUGCGUAUGUGUUU-3′) and scrambled siRNA (5′-CCUCGUGCCGUUCCAUCAGGUAGUU-3′) were used for overall nanocapsule treatment experiments. For in vivo delivery, NRG3 siRNA encapsulated nanoparticles were intravenously injected (1 mg siRNA equiv./kg) into luc-GBM2 (3 × 10^5^ cells) bearing BALB/c nude mice, every other day for a total of five doses over 9 days. The mice were weighed and randomly divided into three groups: Ang-SS-siNRG3, Ang-SS-siScramble, and vehicle.

### Statistics

Data are presented as mean ± SEM based on at least three independent biological replicates, with a minimum of three randomly selected fields analyzed per replicate. Survival distributions were estimated using the Kaplan–Meier method, and observations from patients alive at the last follow-up were treated as censored. Statistical analyses were performed using SPSS version 16 (SPSS Inc., Chicago, IL, USA). For mouse experiments, comparisons between two groups were performed using a two-tailed Student’s t-test, whereas comparisons involving multiple groups were conducted by ANOVA, as appropriate. Survival curves were compared using the log-rank (Mantel–Cox) test. A two-sided P value < 0.05 was considered statistically significant, with individual P values reported in the corresponding figure legends. Differential proteomic and phosphoproteomic analyses were based on nominal P values with Benjamini–Hochberg correction for multiple testing. Nominal P values are displayed as − log10(P) in the plots, while FDR-adjusted q values are indicated by point size. Unless otherwise specified, q < 0.05 was considered statistically significant. Statistical significance is denoted as *P < 0.05, **P < 0.01, and ***P < 0.001.

## Supplementary Information

Supplementary Material 1.

## Data Availability

The data that support the findings of this study are not openly available due to reasons of sensitivity and are available from the corresponding author upon reasonable request.
