# Supplementary material for: Temporally evolving neuronal remodeling via the CTNNB1–NRG3–ERBB4 axis drives therapy resistance in glioblastoma
Source: Mol Biomed. 2026 Nov 6;7:240. doi: 10.1186/s43556-026-00638-7 (PMC13743696; doi:10.1186/s43556-026-00638-7)
Supplement: Supplementary file 1 [file 43556_2026_638_MOESM1_ESM.docx]

**Temporally Evolving Neuronal Remodeling via the CTNNB1–NRG3–ERBB4 Axis Drives Therapy Resistance in Glioblastoma**

Sung Soo Kim^1,†,*^, Ji Yoon Lee^2,†^, Harim Koo^3,†^, Zheng Yuan^4,†^, Kyung-Hee Kim^5,†^, Hyung Joon Kwon^5^, Se Whan Park^5^, Sun Il Choi^5^, Ho-Shin Gwak^5^, Heon Yoo^5^, Young Taek Oh^6^, Yong-Seok Oh^7^, Antonio Iavarone^8^, Seong-Min Park^5,*^, Jason K. Sa^2,*^, Jong Bae Park^9,10,*^

^1^Neuroscience Institution, Lithuanian University of Health Sciences, Kaunas, Lithuania

^2^Department of Biomedical Sciences, Korea University College of Medicine, Seoul, Republic of Korea

^3^Department of Medical Science Convergence, Graduate School of Medical Science, University of Ulsan, Ulsan, Korea

^4^Henan and Macquarie University Joint Centre for Biomedical Innovation, School of Life Sciences, Henan University, Kaifeng, Henan, China

^5^Neuro-Oncology Clinic, National Cancer Center, Goyang, Republic of Korea

^6^Department of Biological Sciences, College of Natural Sciences, Kangwon National University, Chuncheon, Kangwon, Republic of Korea

^7^Department of Brain-Cognitive Science, Daegu-Gyeongbuk Institute of Science and Technology (DGIST), Daegu, Republic of Korea

^8^Sylvester Comprehensive Cancer Center, University of Miami Miller School of Medicine, Miami, FL 33136, USA

^9^Department of Biochemistry and Molecular Biology, School of Medicine, Kyung Hee University, Seoul, Republic of Korea

^10^OmixAI Inc., Seoul, Republic of Korea

^†^These authors contributed equally to this work

^*^Corresponding author:

Sung Soo Kim

ksssea84@gmail.com

Seong-Min Park

lastmhc@ncc.re.kr

Jason K. Sa

jasonksa@korea.ac.kr

Jong Bae Park

jbp@ncc.re.kr

**Keywords**

Glioblastoma; Therapy resistance; NRG3; Temporal; Evolving; Neuronal

**Fig. S1.** Establishment of longitudinal orthotopic PDX models defines biphasic IRP and LRP responses dynamics during anti-VEGF therapy. **a** Representative coronal T2-FLAIR MRI of orthotopic PDX tumors in Balb/c nude mice at the enrollment time point (1 week post-implantation). Mice were stratified by MRI-derived tumor volume to generate size-matched cohorts and then randomized to vehicle (top, n = 71) or anti-VEGF treatment (bottom, n = 71). Images labeled “Excluded” indicate animals whose baseline tumor volumes fell outside the predefined size-matching window and were excluded from the randomized treatment cohorts. **b** Representative LRP GBM2 tumor collected at the 5th treatment time point showing extensive necrosis and HIF-1α–positive pseudopalisading tumor cells, consistent with hypoxia-associated remodeling during late anti-VEGF response. Dashed lines delineate necrotic regions and surrounding pseudopalisading tumor-cell areas. Black arrows indicate corresponding necrotic/pseudopalisading regions shown in the matched low- and high-magnification images. Insets show enlarged views of the boxed regions. Scale bars: 4 mm for low-magnification H&E, and 50 μm for immunofluorescence images.

**Fig. S2.** CTNNB1–NRG3–ERBB4 signaling promotes IRP-associated neuronal reprogramming and GBM cell survival under therapeutic stress. **a** Growth analysis of GBM1-3 cells with CTNNB1 suppression. shRNA-mediated CTNNB1 knockdown reduced growth. Data are presented as mean ± SEM. Statistical significance was determined using one-way ANOVA followed by Tukey’s post hoc test. **b** Effects of NRG3 and ERBB4 silencing on basal growth of parental IRP-derived PDX GBM cells. Parental IRP-derived GBM cells from PDX GBM1, GBM2, and GBM3, without constitutively active CTNNB1-Δ90 expression, were transfected with control siRNA, siNRG3, or siERBB4. Relative cell growth was measured over 3 days. Neither NRG3 nor ERBB4 silencing significantly affected basal cell growth in any of the three parental PDX models compared with the control condition. Data are presented as mean ± SEM from three independent experiments. Statistical significance was assessed using one-way ANOVA with Tukey’s comparison test; all comparisons were not significant (ns).

**Fig. S3.** GBM monoculture control for postsynaptic scaffold-marker staining. **a** Representative immunofluorescence images of IRP-derived GBM2 monocultures stained for Nestin, PSD95/DLG4, and SAP97/DLG1. Nestin was used to identify GSCs, whereas PSD95/DLG4 and SAP97/DLG1 were assessed as postsynaptic scaffold markers. In the absence of mouse cortical neurons, IRP-derived GBM2 monocultures showed weak or diffuse PSD95/DLG4 and SAP97/DLG1 signals with few discrete punctate structures. Scale bar, 50 μm. **b** Representative Western blots of CTNNB1, MAP2, ERBB4, and PSD95 in untreated control GBM cells and cells treated with WNT3A or PRI-724. The untreated condition was included to define basal protein expression and permit direct statistical comparison with the WNT3A- and PRI-724-treated conditions in the Fig. 5c. **c** Effects of PSD95 silencing on basal growth of parental IRP-derived PDX GBM cells. Parental IRP-derived GBM cells from PDX GBM1, GBM2, and GBM3, without PRI-724 treatment, were transfected with control siRNA, siPSD95. Relative cell growth was measured over 3 days. Data are presented as mean ± SEM from three independent experiments. Statistical significance was assessed using one-way ANOVA with tukey’s comparison test; all comparisons were not significant (NS).

**Fig. S4.** Preliminary in vivo screening of WNT/β-catenin pathway inhibitors in the GBM2 orthotopic PDX model. **a** Representative longitudinal T2-FLAIR MRI images of GBM2 orthotopic PDX mice treated with vehicle, VEGF-targeted therapy, ICG-001, LGK974, XAV939, or each inhibitor in combination with VEGF-targeted therapy. MRI monitoring showed that ICG-001, LGK-974, and XAV-939, either alone or in combination with VEGF-targeted therapy, did not produce a clear suppression of tumor progression under the tested conditions. **b** Kaplan–Meier survival analyses of GBM2 orthotopic PDX mice treated with the indicated WNT/β-catenin pathway inhibitors alone or in combination with VEGF-targeted therapy. In contrast to PRI-724, ICG-001, LGK974, and XAV939 did not produce significant survival benefits under the tested dosing conditions. Survival differences were assessed using the log-rank (Mantel–Cox) test. These preliminary screening results supported selection of PRI-724 for subsequent therapeutic validation studies. All comparisons were not significant (NS).

**Materials and Methods**

**Genomic DNA and total RNA extraction and quality check**

Genomic DNA was isolated from cryopulverized tumor tissue using the MagNA Pure 24 Total NA Isolation Kit and MagNA Pure 24 automated extraction system (Roche, Switzerland). Matched germline DNA was extracted from buffy-coat samples using the Maxwell 16 Blood DNA Purification Kit on a Maxwell 16 instrument (Promega, USA). DNA concentration and purity were initially assessed using a NanoDrop 8000 spectrophotometer (Thermo Fisher Scientific, USA). DNA integrity was evaluated by 1% agarose-gel electrophoresis, and double-stranded DNA was quantified using the Qubit dsDNA Broad Range Assay Kit (Thermo Fisher Scientific). Total RNA was extracted from cryopulverized tumor tissue with the RNeasy Mini Kit using a QIAcube automated platform (Qiagen, Germany). RNA concentration and purity were determined using the NanoDrop 8000 spectrophotometer. RNA integrity was subsequently evaluated with the RNA 6000 Nano Kit on an Agilent 2100 Bioanalyzer (Agilent Technologies, USA).

**Whole-exome sequencing**

Whole-exome sequencing (WES) libraries were prepared from genomic DNA. DNA was acoustically fragmented using a Covaris LE220 instrument (Covaris), followed by library construction and exome capture with the Twist Human Core Exome kit (Twist Bioscience, South San Francisco, CA, USA). Captured libraries were sequenced as paired-end reads on an Illumina NovaSeq platform.

**Whole-transcriptome resequencing**

Total RNA concentration was measured using the Quant-iT RiboGreen RNA Assay (Thermo Fisher Scientific). RNA integrity was assessed with RNA ScreenTape on a TapeStation system (Agilent Technologies), and samples with an RNA integrity number (RIN) greater than 7.0 were selected for library preparation. For each sample, 1 μg of total RNA was used to generate a stranded poly(A)-selected mRNA library with the TruSeq Stranded mRNA Library Prep Kit (Illumina), following the manufacturer’s protocol. In brief, polyadenylated transcripts were enriched using oligo(dT) magnetic beads, fragmented, and converted into double-stranded cDNA. After end repair, adapter ligation, and PCR enrichment, indexed libraries were obtained. Library concentration was determined by qPCR using the KAPA Library Quantification Kit for Illumina platforms (KAPA Biosystems, KK4854), and library fragment profiles were evaluated using D1000 ScreenTape (Agilent Technologies, 5067-5582). Pooled indexed libraries were sequenced by Macrogen Inc. on an Illumina NovaSeq platform to generate 100-bp paired-end reads.

**Protein extraction and tryptic Digestion**

Cryopulverized tissue was processed for TMT 11-plex proteome and phosphoproteome profiling. Tissue powder was lysed in 5% SDS/50 mM triethylammonium bicarbonate (TEAB; pH 8.5) and homogenized with an S220 Focused-ultrasonicator (Covaris). Protein extracts were reduced with dithiothreitol and treated with iodoacetamide for cysteine alkylation. The alkylation reaction was quenched before adjustment of the lysates to final concentrations of 5% SDS and 1.2% phosphoric acid. Samples were diluted with 90% methanol prepared in 100 mM TEAB and processed using S-Trap micro spin columns (Protifi, Huntington, NY). On-column proteolysis was carried out with mass-spectrometry-grade trypsin/Lys-C (Promega) at 47°C for 3 h. Recovered peptides were vacuum-dried and desalted on C18 spin columns (Thermo Fisher Scientific) before downstream TMT labeling and LC–MS/MS analysis.

**TMT 11-plex labeling**

Desalted peptides were reconstituted in 100 mM triethylammonium bicarbonate (TEAB) and labeled with TMT 11-plex reagents. Each TMT reagent was added to the corresponding peptide sample, followed by incubation for 1 h at room temperature. Labeling reactions were terminated by adding 8 μL of 5% hydroxylamine and incubating the samples for an additional 15 min. The 11 differentially labeled peptide samples were then combined and dried in a vacuum concentrator.

**Peptide fractionation by Mid-pH reverse phase liquid chromatography**

The pooled TMT 11-plex-labeled peptide mixture was fractionated by high-pH reversed-phase HPLC using an Agilent 1260 Infinity system (Agilent Technologies, Palo Alto, CA, USA) equipped with an XBridge C18 column (4.6 × 250 mm, 130 Å pore size, 5 μm particle size). Mobile phase A consisted of 10 mM triethylammonium bicarbonate (TEAB) in water (pH 7.5), whereas mobile phase B comprised 10 mM TEAB in 90% acetonitrile (pH 7.5). Peptides were eluted at 500 μL/min using the following gradient: 0% B for 15 min; 0–5% B over 10 min; 5–35% B over 60 min; 35–70% B over 15 min; 70% B for 10 min; and 70–0% B over 10 min. Fractions were collected at 1-min intervals between 15 and 110 min, producing 96 initial fractions. These fractions were concatenated into 24 non-adjacent peptide pools. After C18 desalting (Thermo Fisher Scientific), 5% of each pooled fraction was reserved, vacuum-dried, and used for global proteome analysis. The remaining 95% was further combined into 12 fractions, dried under vacuum, and subjected to phosphopeptide enrichment.

**Phosphopeptide enrichment**

Phosphopeptides were enriched by Fe³⁺-immobilized metal affinity chromatography (Fe³⁺-IMAC). Ni-NTA magnetic agarose beads (Qiagen, Valencia, CA, USA) were converted to Fe³⁺-NTA beads by sequential treatment with 100 mM EDTA (pH 8.0) and 10 mM aqueous FeCl₃. Each treatment was performed for 30 min with end-over-end rotation, with deionized-water washes between steps. The resulting Fe³⁺-charged beads were suspended in acetonitrile/methanol/0.01% acetic acid (1:1:1, v/v/v) and distributed into microcentrifuge tubes. Fractionated peptides were dissolved in loading buffer containing 80% acetonitrile and 0.1% trifluoroacetic acid (TFA). After equilibration with the same buffer, Fe³⁺-NTA beads were incubated with the peptide samples for 30 min using end-over-end rotation. The unbound fraction was retained and dried for later analysis. Beads were washed with loading buffer to remove non-specifically bound peptides. Bound phosphopeptides were recovered with acetonitrile/2.5% ammonium hydroxide/2 mM phosphate buffer (1:1:1, v/v/v), immediately acidified using 10% TFA, and dried in a vacuum concentrator.

**LC-MS/MS analysis**

TMT-labeled peptides prepared for global proteome and phosphoproteome analysis were resuspended with 0.1% formic acid in water, separated, and analyzed on a Q Exactive HF-X Hybrid Quadrupole-Orbitrap mass spectrometer (Thermo-Fisher) coupled with an Ultimate 3000 RSLCnano system (Thermo-Fisher). The peptides were loaded onto a trap column (100 µm × 2 cm) packed with Acclaim PepMap100 C18 resin on which loaded peptides were eluted with a linear gradient from 5% to 28% solvent B (0.1% formic acid in ACN) for 110 min at a flow rate of 300 nl/min. The eluted peptides, separated by the analytical column (EASY-Spray column, 75 μm × 50 cm; Thermo-Fisher), were sprayed into the nano-ESI source with an electrospray voltage of 2.1 kV. The Q Exactive HF-X Orbitrap mass analyzer was operated in the top 20 data-dependent method. Full MS scans were acquired over the range m/z 350 to 1,800 with a mass resolution of 60,000 (at m/z 200). The AGC target value was 3.00E+06. The twenty most intense peaks with a charge state ≥ 2 were fragmented in the higher energy collisional dissociation (HCD) collision cell with a normalized collision energy of 28, and tandem mass spectra were acquired in the Orbitrap mass analyzer with a mass resolution of 15,000 at m/z 200. Database searching of all raw data files was performed in Proteome Discoverer 2.2 software (Thermo-Fisher). SEQUEST-HT was used for database searching against the SwissProt human database. Database searching against the corresponding reverse database was also performed to evaluate the false discovery rate (FDR) of peptide identification. The database searching parameters included a precursor ion mass tolerance of 10 ppm, a fragment ion mass tolerance of 0.08 Da, a fixed modification for carbamidomethyl cysteine (+57.021Da/C) and variable modifications for methionine oxidation (+15.995 Da/M). We obtained an FDR of less than 1% at the peptide level and filtered it with high peptide confidence.

**Database search and processing**

Raw mass spectrometry files were converted to open mzML format using the msconvert utility of the ProteoWizard software suite. The mzML spectral data were mapped to the human UniProt database (UP000005640.fasta.gz). FragPipe (ver.17.0), including MSFragger, Philosopher and TMT-integrator program. All identified proteins had an FDR of 1%, which was calculated at the peptide level. For the global proteome, the search parameters allowed for tryptic specificity of up to two missed cleavages, with carbamidomethyl-modifications of cysteine as a fixed modification and oxidation of methionine as a dynamic modification. The mass search parameters for -1, 0, +1, +2, and +3 ions included mass error tolerances of 20 ppm for precursor ions. For the phosphoproteome, the search parameters allowed for tryptic specificity of up to two missed cleavages, with modified serine, tyrosine, and threonine as variable modifications. The protein expression was quantified with the isobaric TMT 11 option and normalized to the relative expression ratio against the global reference pool of each TMT 11 set. To correct sample loading disparities, data were normalized using the median centering method across total proteins or phosphorylation sites. Batch corrections were applied to global proteome and phosphoproteome abundance data to remove the technical differences between different TMT runs before performing advanced analysis. The batch effect was evaluated by PCA and corrected with ComBat package, an R library with tumor/normal status adjustment, was applied. To impute missing values, the DreamAI package was used to perform an imputation of the proteins and phosphopeptides with a missing rate of less than 50%.

**Validation with transcriptomic data for human**

Transcriptomic analysis was performed using RNA-seq data obtained from tumor samples of glioblastoma patients before and after treatment with Bevacizumab plus irinotecan (GSE79671). FPKM was used for statistical analysis.

**Pathway enrichment analysis**

Functional enrichment of differentially expressed proteins was evaluated using the clusterProfiler R package. Gene Ontology (GO) terms and Kyoto Encyclopedia of Genes and Genomes (KEGG) pathways were tested separately for proteins altered in initial-response phase (IRP) and late-response phase (LRP) GBM models relative to the control group.

**Mutation analysis**

Paired-end sequencing reads in FASTQ format were mapped to the human reference genome (GRCh37/hg19) using the Burrows–Wheeler Aligner. The resulting alignment files were processed through coordinate sorting, duplicate marking/removal, local indel realignment, and base-quality score recalibration using SAMtools, Picard, and the Genome Analysis Toolkit (GATK). Somatic variants were identified with MuTect2. Known polymorphic sites from the 1000 Genomes Project, gnomAD, and dbSNP databases were incorporated during variant filtering and interpretation. Functional consequences of retained variants were annotated using Variant Effect Predictor (VEP).

**Pathway enrichment score analysis**

Single-sample gene set enrichment analysis (ssGSEA), implemented in the GSVA R package, was used to calculate pathway-enrichment scores for each sample. Functional pathway activity was then compared among vehicle-treated, initial-response phase (IRP), and late-response phase (LRP) GBM models. Gene sets were obtained from the curated collection c2.all.v7.1.symbols.gmt (MSigDB).

**PPI network formation**

Protein–protein interaction (PPI) information was retrieved from the STRING database. Interactions were filtered using a minimum confidence score of 0.400, and molecular-action evidence was used to define the interaction edges. Proteins associated with pathways specific to the initial-response phase (IF) or late-response phase (LF) were selected for network analysis. The filtered interaction dataset was imported into Cytoscape version 3.8.2 to generate and visualize the PPI network.

**Gene set enrichment analysis (GSEA)**

Gene set enrichment analysis (GSEA) was performed with the Java implementation of GSEA (version 4.3.2; Broad Institute). Normalized gene-expression data were ranked according to the signal-to-noise statistic and analyzed against the curated gene-set collection, c2.all.v7.1.symbols.gmt. Enrichment significance was assessed using 1,000 gene-set permutations. Nominal P values were calculated by GSEA, and multiple-testing adjustment was performed with the Benjamini–Hochberg procedure. Gene sets with a false-discovery-rate-adjusted q value of <0.05 were regarded as significantly enriched.

**Inflection-point and segmented tumor-growth analysis**

Longitudinal MRI-derived tumor volumes were analyzed using the fourth time point as the shared kinetic breakpoint. The pre-breakpoint segment encompassed the first through fourth time points and represented the IRP trajectory, whereas the post-breakpoint segment extended from the fourth to the fifth time point and represented the emerging LRP trajectory. Thus, the fourth time point represented both the final IRP measurement and the onset of the LRP growth trajectory. The pre-breakpoint growth slope (βpre) was estimated by linear regression using tumor volumes measured from the first through fourth time points. Because the post-breakpoint segment contained the fourth and fifth measurements, the post-breakpoint growth slope (βpost) was calculated as: V=Volume, t=phase

The increase in growth rate across the fourth-time-point breakpoint was quantified as the ratio of the post-breakpoint to pre-breakpoint slopes:

A slope ratio of Rslope≥2.5 was used as the operational criterion for identifying the accelerated LRP trajectory. Therefore, the ≥2.5-fold criterion represented an increase in tumor-growth slope across the fourth-time-point breakpoint and not a direct ratio of tumor volumes measured at the fifth and fourth time points.

Based on this analysis, the pre- and post-breakpoint slopes were 5.0 and 105.0 for GBM1, 5.0 and 85.0 for GBM2, and 3.67 and 145.0 for GBM3, respectively. The corresponding slope ratios were 21.0, 17.0, and approximately 39.5, each exceeding the operational ≥2.5-fold criterion. Local growth acceleration centered on the fourth time point was additionally evaluated using a discrete second-difference analysis. Because the MRI measurements were obtained at equally spaced intervals, the local second difference was calculated as:

At the fourth time point, this corresponded to:

Positive values indicated local acceleration in tumor growth, whereas negative values indicated deceleration. A marked positive increase in the second difference centered on the fourth time point was observed across GBM1–3, supporting its designation as the operational IRP-to-LRP kinetic inflection point. The slope-ratio and curvature analyses were performed to define and characterize the growth-kinetic transition centered on the fourth time point. Downstream transcriptomic, proteomic, histological, and functional analyses were subsequently performed using samples assigned to the corresponding imaging-defined response states.

**BLI Images**

Luciferase-expressing GBM cells were generated by lentiviral transduction with a reporter vector encoding firefly luciferase under the control of the promoter. Lentiviral particles were produced in 293T cells using the PSPAX2 and PMD2.G packaging system. GBM cells were exposed to the viral supernatant at a multiplicity of infection in the presence of polybrene. Reporter-expressing cells were selected using puromycin for 3days and subsequently maintained under the same culture conditions as the corresponding parental cells. Luciferase activity was confirmed after exposure to D-luciferin using IVIS Lumina XRMS Series III system before intracranial implantation. Cell viability was confirmed before implantation, and the number of implanted luciferase-expressing cells used in each experiment is provided in the corresponding figure legend. Longitudinal bioluminescence imaging was performed using an IVIS Lumina XRMS Series III system (PerkinElmer). Mice bearing orthotopic luciferase-expressing GBM tumors were imaged twice weekly beginning one week after implantation. Before image acquisition, mice were administered D-luciferin at 10 mg/kg by IP and anesthetized using isoflurane 1%. Images were acquired 1 min after D-luciferin administration. The photons emitted from luciferase-expressing tumor cells were quantified using Living Image software. A pseudocolor image representing light intensity (blue = least intense and red = most intense) was generated and superimposed over the corresponding grayscale reference image.

For quantitative analysis, a region of interest (ROI) was placed over the cranial bioluminescent signal corresponding to the orthotopic tumor. Tumor-associated bioluminescence was quantified as the total photon flux within the ROI and expressed as photons per second. Background photon flux was measured using a tumor-free reference region and subtracted where applicable. Identical acquisition parameters and consistent ROI placement criteria were used across treatment groups and longitudinal time points. Consistent ROI-selection criteria and image-acquisition settings were applied across treatment groups and longitudinal time points. ROI-based photon flux was used to longitudinally assess viable tumor burden. Mice were euthanized when they met predefined humane endpoint criteria, including neurological deficits, >20% body-weight loss, or signs of severe distress. GBM-Luc cells were generated by transduction with a luciferase-expressing vector to enable longitudinal in vivo monitoring of tumor burden.

**Isolation of mouse cortical neurons and coculture**

Primary cortical neurons were prepared from embryonic day 15 (E15) mouse embryos with minor modifications to a previously described procedure [17]. Cerebral cortices were isolated, incubated with papain in HBSS containing DNase at 37°C for 40 min, and washed three times with minimum essential medium (MEM). The tissue was then mechanically dissociated in MEM to generate a single-cell suspension. After centrifugation, cells were resuspended in prewarmed Neurobasal medium supplemented with GlutaMAX and B27 and seeded onto poly-L-lysine-coated culture dishes. Neuronal cultures were maintained for 10 days in vitro (DIV). Half of the culture medium was replaced with fresh, prewarmed neuronal medium on DIV 3, 7, and 10. For neuron–GBM co-culture experiments, cortical neurons were co-cultured with GBM cells for 3 days in DMEM containing 10% fetal bovine serum and 1% penicillin–streptomycin. Co-cultures were subsequently processed for confocal microscopy. Puncta were quantified. Immunofluorescence images were analyzed using Zeiss 780 laser scanning confocal/ImageJ. For GBM-cell-restricted analysis, Nestin-positive regions were used to define GBM tumor/stem-like cell regions of interest (ROIs). Quantification was restricted to Nestin-positive GBM-cell-defined regions and was not performed across the entire mixed co-culture field. For SAP97/DLG1 analysis, discrete puncta were quantified in standardized 200 × 110 μm ROIs. SAP97/DLG1-positive puncta were identified using the particle analysis function in Zeiss 780 laser scanning confocal/ImageJ after thresholding of the SAP97/DLG1 channel. Round punctate structures with a diameter of ≤6 μm were counted as discrete puncta, whereas diffuse staining and larger nonspecific aggregates were excluded. Puncta overlapping with Nestin-positive GBM-cell regions were included in the final quantification. This analysis was used to estimate postsynaptic scaffold-marker positivity within GBM-cell-defined regions rather than overall signal intensity across the mixed co-culture field. For each condition, quantification was performed across randomly selected fields from independent cultures/experiments. The number of fields, biological replicates, and independent experiments are indicated in the corresponding figure legends. Image analysis was performed using the same thresholding and particle-size criteria across all groups. Data are presented as mean ± SEM. Statistical significance was assessed using one-way ANOVA followed by Tukey’s multiple-comparison test, unless otherwise indicated. Quantification was performed across three independent biological replicates, with at least three randomly selected fields analyzed per replicate.

**Cryopulverization of tissue samples**

Tumor-containing regions were macrodissected to minimize contamination by adjacent non-neoplastic brain tissue. Only specimens with an estimated tumor-cell content of >90% (±5%) were included in subsequent proteogenomic analyses. Following MRI assessment, mouse brains were collected and allocated for either histopathological evaluation or cryogenic tissue processing. Macrodissected frozen tumor samples were weighed and briefly rinsed with ice-cold phosphate-buffered saline (PBS) to remove residual blood. Tissues were placed in tissueTUBE vessels (Covaris, Woburn, MA), snap-frozen in liquid nitrogen, and pulverized using a cryoPREP Tissue Disruption System (CP02; Covaris). The resulting tissue powder was aliquoted into 10–20 mg portions for DNA, RNA, and protein extraction.

**Lentivirus production and infection**

Lentiviral particles encoding CTNNB1 Δ90 (Addgene plasmid #36985) or shCTNNB1 (Addgene plasmid #19761) were generated in HEK293T cells. Briefly, 6 × 10⁶ HEK293T cells were seeded in 100-mm dishes 1 day before transfection. Cells were transfected with the relevant lentiviral transfer vector together with the packaging plasmid psPAX2 and envelope plasmid pMD2.G, using Lipofectamine 2000 (Invitrogen). For each dish, 4.5 μg of the transfer plasmid, 3 μg of psPAX2, and 1.5 μg of pMD2.G were used. Culture medium was replaced 6 h after transfection. Viral supernatants were collected 48 h later, concentrated using Lenti-X Concentrator (Clontech), and used to transduce target cells in the presence of polybrene (10 μg/mL).

**Culture of patient-derived GBM cells and established cell lines**

Four patient-derived glioblastoma stem cell (GSC) cultures, designated GBM1–GBM4, were established as reported previously [8–17]. GSCs were propagated in serum-free DMEM/F-12 medium containing B27 supplement (Invitrogen), recombinant human EGF (10 ng/mL; R&D Systems), basic FGF (5 ng/mL; R&D Systems), and 1× penicillin–streptomycin. GBM2 cells and HEK293T cells were cultured in DMEM supplemented with 10% fetal bovine serum (HyClone). All cultures were maintained at 37°C in a humidified atmosphere containing 5% CO₂. Cell-line identity was verified by short tandem repeat profiling. Cultures were routinely screened for mycoplasma contamination at 6-month intervals and before key in vitro or in vivo experiments.

**Tissue processing and in vivo drug treatment**

At the designated experimental endpoint, brains were collected for histological or molecular analysis. For histological assessment, harvested brains were fixed in 4% paraformaldehyde. For multi-omics profiling, tumor tissues from three individual mice per group were processed separately to preserve biological replicates and to obtain adequate material for each downstream assay. Tumor-enriched regions (>90% ± 5% tumor cells) were macrodissected under MRI guidance and snap-frozen for parallel whole-exome/RNA sequencing and LC–MS/MS-based proteomic/phosphoproteomic analyses. Bevacizumab (10 mg/kg; Roche) was delivered by intraperitoneal injection every 2 days, for a total of four our or five doses, unless mice reached a predefined humane endpoint earlier. ICG-001 (50 mg/kg; Selleck Chemicals), LGK (3 mg/kg; Selleck Chemicals), XAV-939 (50 mg/kg; Selleck Chemicals), and PRI-724 (60 mg/kg; Selleck Chemicals) were administered orally once daily until the scheduled sampling time or humane endpoint, as specified for each experiment. Humane endpoints were defined as a loss of more than 20% of baseline body weight or severe neurological impairment, including marked lethargy, ataxia, or seizures.

**Cell proliferation assays**

The luminescence of viable cells was detected using s CellTiter-Glo Luminescent Cell Viability Assay Kit according to the manufacture’s protocol (Promega). The CellTiter-Glo Luminescent Cell Viability Assay is a homogenous method of determining the number of viable cells in culture based on quantitation of the ATP present, which signals the presence of metabolically active cells. The luminescence signal was detected by a SpectraMax L Microplate Reader (Molecular Device) according to the manufacturer’s protocol. The results were analyzed and visualized using Microsoft Excel 2019.

**Reverse Transcription-Polymerase Chain Reaction**

Semiquantitative and real-time RT-PCR were performed to determine mRNA levels as previously described. Total RNA was isolated from cells using TRIzol reagent (Invitrogen) according to the manufacturer’s instructions. Total RNA (500 ng) was used as a template to synthesize cDNA using M-MLV reverse transcriptase (Promega). Real-time RT-PCR analysis was performed on a LightCycler 480II real-time detection system (Roche) using LightCycler 480 SYBR Green I Master Mix (Roche). The expression levels of target genes were normalized to that of the input signal. Semiquantitative RT-PCR products were analyzed on a 1% agarose gel. The PCR primers are shown as follows: NRG3, sense 5′-ACGACGACATATTCCACAGAGC-3′ and antisense 5′-CCGGTCAGGGTTTCGATCAC-3′; MAP2, sense 5′CGAAGCGCCAATGGATTCC-3′ and antisense 5′-TGAACTATCCTTGCAGACACCT-3′; ERBB4, sense 5′-GCCTCTGGAGAATTTACGCAT-3′ and antisense 5′-GGGTTCCGAACAATATCTTGCC-3′;

**Imaging equipment and analysis**

Confocal imaging was carried out on a Zeiss 780 laser scanning confocal scan head operating on an Axiovert Z1 inverted microscope equipped with a 20x, 40x objective. High-speed superresolution imaging was carried out on a Zeiss 880 Airyscan confocal with a 63x 1.4 oil NA objective. Image analysis was performed using ZEN Black software for Airyscan processing, which involves signal integration from the 32 separate subresolution detectors in the Airyscan detector and subsequent deconvolution of this integrated signal. All samples included in the same quantitative comparison were imaged using identical laser power, detector gain, exposure, objective magnification, pixel resolution, and image-processing settings. Viable tumor regions were selected using consistent anatomical and morphological criteria, and necrotic, hemorrhagic, folded, or otherwise artifactual areas were excluded. At least three non-overlapping fields were analyzed from each independent tumor or biological replicate. Values obtained from the analyzed fields were averaged to generate one value for each independent tumor or biological replicate; individual fields were not treated as independent biological replicates.Image quantification was performed using ZEN or ImageJ software. Background fluorescence was subtracted uniformly, and identical thresholding parameters were applied to all images within an experiment.
